# Insulin Resistance in Human iPS Cells Reduces Mitochondrial Size and Function

**DOI:** 10.1038/srep22788

**Published:** 2016-03-07

**Authors:** Alison M. Burkart, Kelly Tan, Laura Warren, Salvatore Iovino, Katelyn J. Hughes, C. Ronald Kahn, Mary-Elizabeth Patti

**Affiliations:** 1 Integrative Physiology and Metabolism Research Division, Joslin Diabetes Center and Harvard Medical School, Boston, MA, 02215, USA.

## Abstract

Insulin resistance, a critical component of type 2 diabetes (T2D), precedes and predicts T2D onset. T2D is also associated with mitochondrial dysfunction. To define the cause-effect relationship between insulin resistance and mitochondrial dysfunction, we compared mitochondrial metabolism in induced pluripotent stem cells (iPSC) from 5 healthy individuals and 4 patients with genetic insulin resistance due to insulin receptor mutations. Insulin-resistant iPSC had increased mitochondrial number and decreased mitochondrial size. Mitochondrial oxidative function was impaired, with decreased citrate synthase activity and spare respiratory capacity. Simultaneously, expression of multiple glycolytic enzymes was decreased, while lactate production increased 80%. These perturbations were accompanied by an increase in ADP/ATP ratio and 3-fold increase in AMPK activity, indicating energetic stress. Insulin-resistant iPSC also showed reduced catalase activity and increased susceptibility to oxidative stress. Thus, insulin resistance can lead to mitochondrial dysfunction with reduced mitochondrial size, oxidative activity, and energy production.

Insulin resistance is a key component of type 2 diabetes (T2D) pathophysiology and an early marker and predictor of diabetes risk^1^, occurring several decades before hyperglycaemia develops. Due to the difficulty in accessing important tissues for study in humans, the specific molecular mechanisms responsible for common forms of insulin resistance remain unknown but likely include genetic, developmental, and environmental contributions.

Insights into human insulin resistance have been provided by rare syndromes due to insulin receptor (INSR) mutations, such as Donohue syndrome and type A insulin resistance^2^,^3^,^4^,^5^,^6^,^7^. Clinically, these syndromes share several features, including acanthosis nigricans, hyperandrogenism, and severe insulin resistance. In addition, patients with Donohue syndrome display reduced body weight and postnatal growth. Recent data have also demonstrated that humans with *INSR* mutations may have abnormalities in mitochondrial function, as indicated by decreased phosphocreatine recovery in muscle after exercise^8^. While skin fibroblasts derived from these individuals have elucidated key information about the structure and function of the insulin receptor and its downstream signalling pathways^9^, these cells are already terminally differentiated and are poorly responsive to insulin. To address these limitations, we have utilised induced pluripotent stem cells (iPSC) derived from these patients as a new model system to identify differentiation-independent and cell autonomous molecular drivers of insulin resistance. These cells also allow us to identify mechanisms by which insulin resistance modulates stem cell function and metabolism.

Both human and animal studies highlight key links between insulin resistance and energetic defects^10^,^11^. For example, *in vivo* magnetic resonance spectroscopy studies in patients with T2D demonstrate reduced ATP synthesis^12^,^13^,^14^,^15^ and tricarboxylic acid (TCA) cycle flux^16^. Moreover, muscle biopsies from individuals with T2D and obesity-linked insulin resistance show reductions in nuclear-encoded mitochondrial gene expression^17^,^18^, mitochondrial DNA (mtDNA) levels^19^, and oxidative phosphorylation (OXPHOS) complex activity^20^. Conversely, interventions such as exercise, caloric restriction, and weight loss typically improve mitochondrial activity and insulin sensitivity in parallel^21^,^22^,^23^,^24^.

We have previously shown that insulin resistance affects stem cell function through decreased cell proliferation; however, it remains unknown whether insulin resistance can also affect metabolism in these cells. To test the hypothesis that insulin resistance can modulate stem cell metabolism, we utilised our unique collection of iPSC from patients with severe insulin resistance (IR-Mut) and observed several metabolic defects including decreased mitochondrial size and functional capacity.

## Results

**Figure 1:**
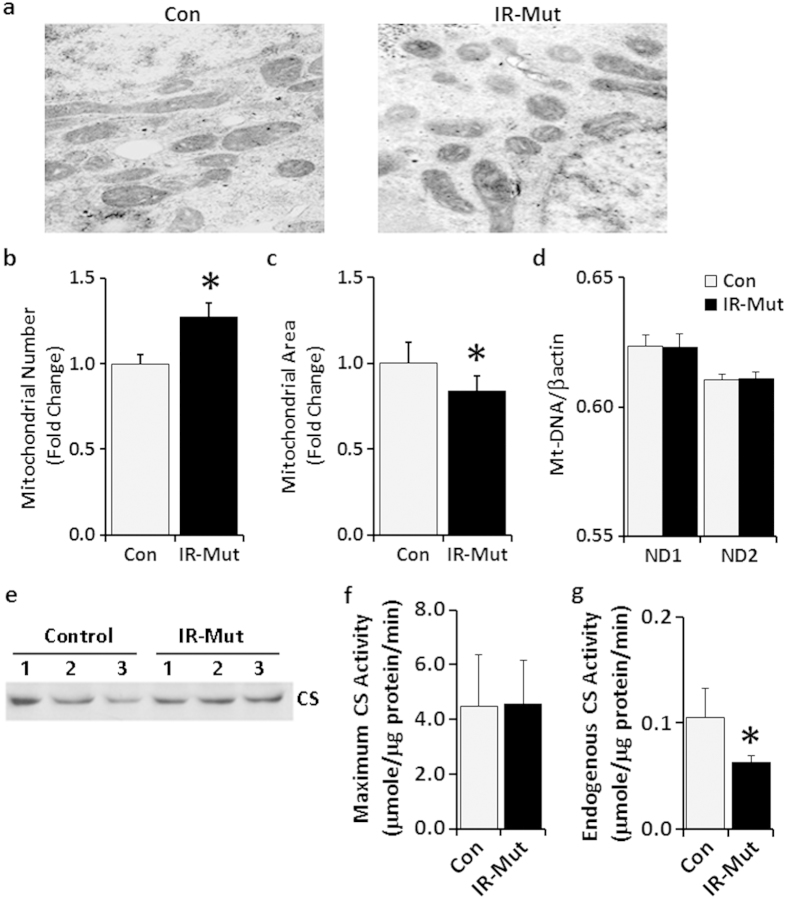
Decreased mitochondrial area and CS activity in IR-Mut iPSC. (**a**) Representative electron micrographs of control and IR-Mut iPSC. (**b**) Quantification of the number of mitochondria relative to total cellular area. Images at 4500X; 9 micrographs/cell line. (**c**) Quantification of the area of mitochondria relative to total cellular area. Images at 34000X; 5 micrographs/cell line. (**d**) Mitochondrial DNA content, as determined by qRT-PCR using primers for *mt-ND1* and *mt-ND2*, with nuclear beta actin as control (n = 3). (**e**) Expression of CS protein by representative western blot. (**f,g)** Assay for maximal (**f**) and endogenous (**g**) citrate synthase activity measured as μmole/min and normalised to cellular protein (n = 3). *indicates p < 0.05 for IR-Mut vs. control.

iPS cells from control individuals and patients with insulin resistance were generated and characterised previously^25^. Clinical information is presented in Supplementary Table S1. To determine if mitochondrial number or structure was altered by insulin resistance, electron microscopy analysis was performed. IR-Mut iPSC had a 22% increase in mitochondrial number (p = 0.02; Fig. 1a,b), but a 16% decrease in mitochondrial area (p = 0.04; Fig. 1a,c and Supplementary Fig. S1). This increase in number of smaller mitochondria was associated with increased expression of mitochondrial fission factor (*MFF*) and inverted formin 2 (*INF2*), which both promote mitochondrial fission, whereas expression of genes regulating mitochondrial fusion were unchanged between control and IR-Mut iPS (Supplementary Fig. S2A,B). mtDNA content (Fig. 1d), MitoTracker Green staining (Supplementary Fig. S3), and mRNA expression of mitochondrial transcription factor A (*TFAM*), which is also a regulator of mtDNA replication (Supplementary Fig. S4A), were all similar between control and IR-Mut iPSC. Likewise, citrate synthase (CS) protein, a marker for mitochondrial mass, and CS maximal activity, examined in the presence of exogenous oxaloacetate, did not differ between control and IR-Mut iPSC (Fig. 1e,f), suggesting that total mitochondrial mass was similar in the control and insulin-resistant iPSC, despite the changes in mitochondrial size and shape. Interestingly, in the absence of exogenous substrate, endogenous CS activity was decreased by 40% in insulin-resistant iPSC (p = 0.04; Fig. 1g). Collectively, these data indicate similar mitochondrial mass but reduced CS activity, potentially due to substrate limitation.

**Figure 2:**
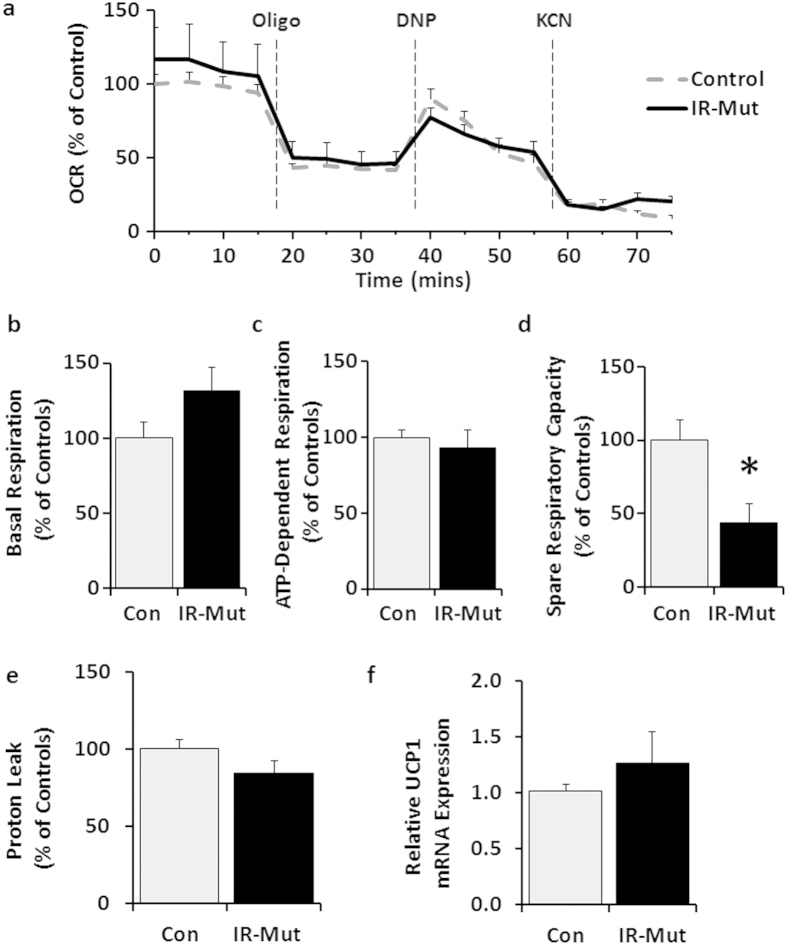
Decreased spare respiratory capacity in IR-Mut iPSC. (**a**) Bioenergetic profile for oxygen consumption rate (OCR) of control and IR-Mut iPSC assessed at baseline and following sequential addition of oligomycin (10 μg/ml), DNP (10 μM) and KCN (50 nM). OCR was analysed at 5-minute intervals and normalised by DNA (n = 3). (**b**) Basal OCR was calculated from the last measurement before oligomycin addition minus non-mitochondrial respiration. (**c**) ADP-dependent respiration calculated from last measurement before oligomycin minus minimum measurement after oligomycin. (**d**) Spare respiratory capacity calculated from maximum measurement after DNP minus last measurement before oligomycin. (**e**) Proton leak calculated from minimum measurement after oligomycin minus non-mitochondrial respiration. (**f**) Relative mRNA expression of uncoupling protein 1, as assessed by qRT-PCR. Data are normalised to *36B4* and expressed relative to control (n = 3). *p < 0.05 for IR-Mut vs. control.

To determine if the change in mitochondrial structure and citrate synthase activity was paralleled by a change in oxidative metabolism, cellular oxygen consumption was measured under basal conditions and after treatment with compounds that block different mitochondrial proteins to interrogate several components of mitochondrial oxidative function (Fig. 2a). There was a trend for increased basal respiration (p = 0.08) in insulin-resistant iPSC, but no difference in ATP-dependent respiration (Fig. 2b,c), calculated by the change in oxygen consumption rate (OCR) upon addition of the ATP synthase inhibitor oligomycin. Treatment with the uncoupler 2,4-dintrophenol (DNP) induced maximum respiration to a similar level in control and IR-Mut iPSC (Fig. 2a). However, spare respiratory capacity, i.e. ability to respond to increased energy demand, was decreased by 56% in the IR-Mut cells (p = 0.01; Fig. 2d), calculated by the difference between maximal and basal respiration. The decreased capacity was not due to uncoupling, as evidenced by similar proton leak (Fig. 2e) and unchanged expression of uncoupling protein 1 (*UCP1*) (Fig. 2f).

**Figure 3:**
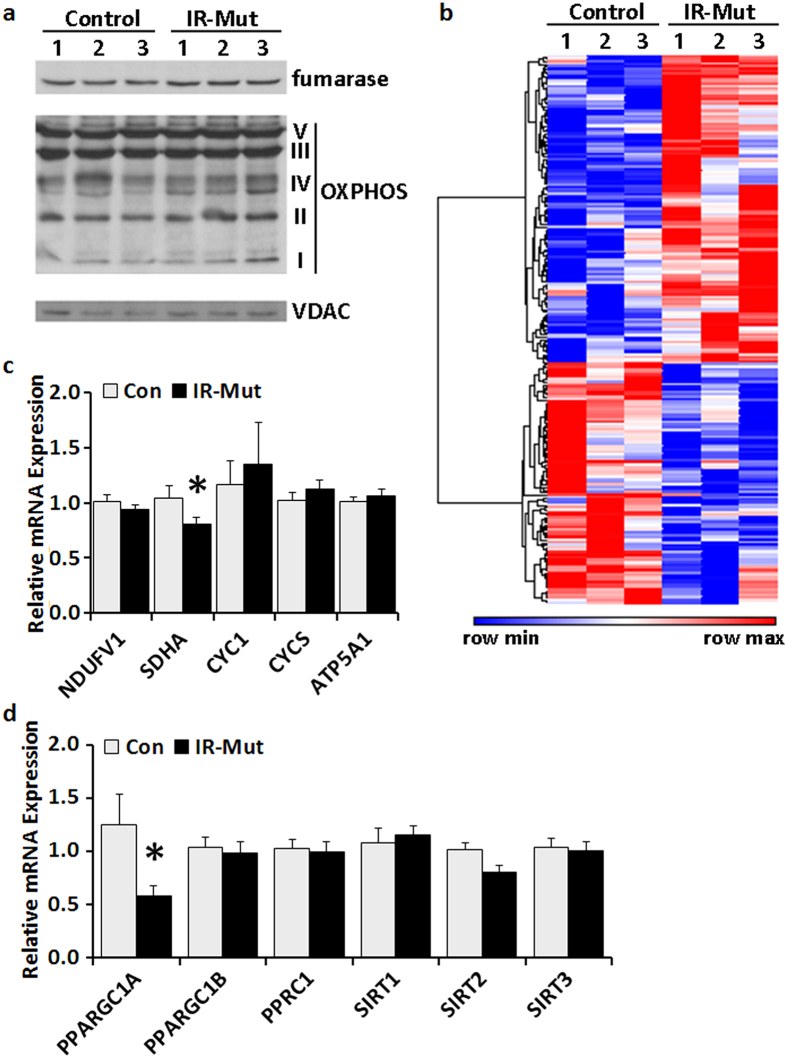
IR-Mut iPSC have decreased PGC1α expression. (**a**) Representative western blots showing protein expression of fumarase and key enzymes in OXPHOS. VDAC was used as a loading control (n = 3). (**b**) Heat map of differentially expressed genes (p < 0.05) related to mitochondria using ontology terms “mitoc*” as determined by microarray analysis, with red indicating increased expression and blue indicating decreased expression. (**c**) Relative mRNA expression of selected nuclear-encoded mitochondrial genes, as assessed by qRT-PCR. Data are normalised to *36B4* and expressed relative to control (n = 3). (**d**) Relative mRNA expression of selected genes regulating mitochondrial biogenesis and metabolism, as assessed by qRT-PCR. Data are normalised to *36B4* and expressed relative to control (n = 3). *indicates p < 0.05 for IR-Mut vs. control.

We next examined expression of mitochondrial proteins, including OXPHOS subunits, as a possible cause of this perturbed oxidative metabolism. Western blot analysis revealed no difference between control and insulin-resistant iPSC in representative components of the TCA cycle (fumarase) or OXPHOS complexes (NADH dehydrogenase [ubiquinone] 1 beta subcomplex subunit 8 (complex 1), complex II subunit 30 kDa, complex III subunit core 2, complex IV subunit I, and ATP synthase subunit alpha; Fig. 3a). Gene expression data from prior microarray analysis^25^ were queried to examine the expression of genes that were significantly altered in IR-Mut cells and also annotated with mitochondrial ontology terms (p < 0.05; Fig. 3b, Supplementary Table S2), with 150 of 1312 mitochondrial ontology genes significantly changed. Expression of 12 genes related to oxidative phosphorylation was significantly altered (with 8 increased and 4 decreased), including succinate dehydrogenase complex, subunit A (*SDHA*) and several ETC subunits. Furthermore, 6 genes involved in stress response were downregulated, while 17 genes related to mitochondrial ribosomes and protein translation were significantly increased. qPCR confirmed a small but significant decrease in *SDHA* (Fig. 3c), while representative genes in other electron chain complexes were unchanged. Interestingly, expression of the mitochondrial regulatory coactivator peroxisome proliferator-activated receptor gamma, coactivator 1 alpha *PPARGC1A* (encoding PGC1α) was reduced by 54% in IR-Mut iPSC (p < 0.001; Fig. 3d); other transcriptional regulators of mitochondria and metabolism, including *PPARGC1B, PPRC1*, *SIRT1*, *SIRT2*, and *SIRT3* (Fig. 3d) and *GABPA, GABPB1*, *GABPB2*, *RB1*, *E2F1, E2F4*, *ESRRA*, and *ESRRG* (Supplementary Fig. S4A), were unchanged.

**Figure 4:**
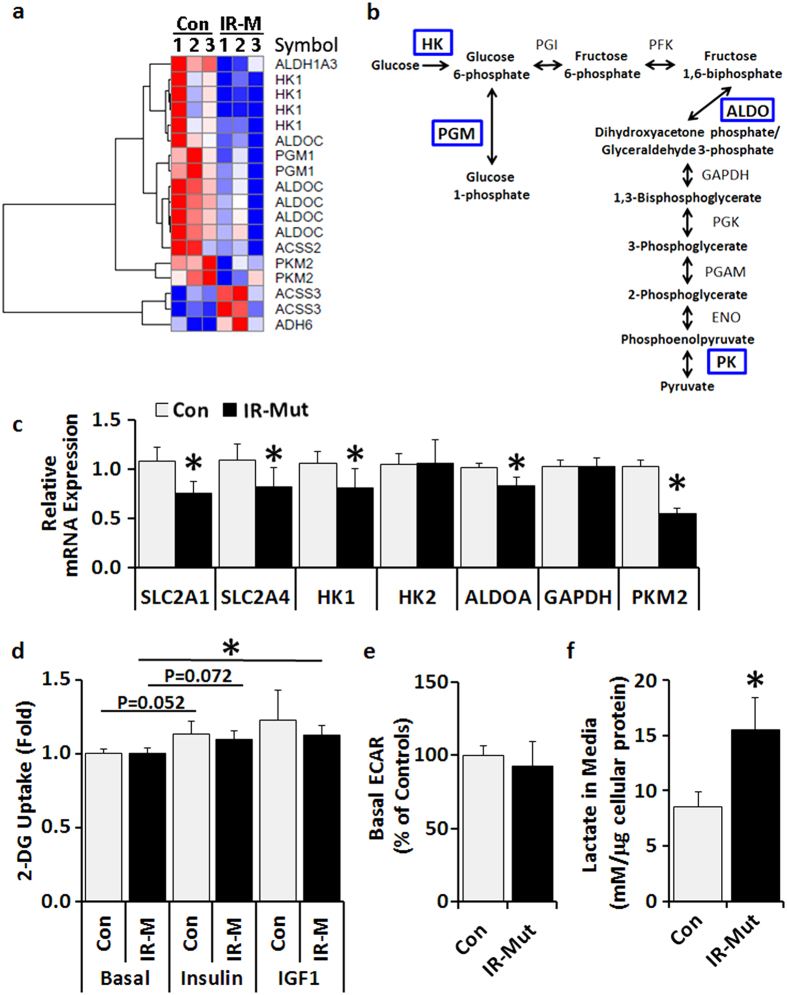
IR-Mut iPSC have increased extracellular lactate. (**a**) Heat map of differentially expressed genes (nominal p < 0.05) related to glucose metabolism based on KEGG glycolysis/gluconeogenesis gene set, as determined by microarray analysis, with red indicating increased expression and blue indicating decreased expression. (**b**) Glycolysis pathway schematic of enzymes with significantly decreased mRNA expression in IR-Mut iPSC (indicated in blue). (**c**) Relative mRNA expression of glucose metabolism genes, as assessed by qRT-PCR. Data are normalised to *36B4* and expressed relative to control cells (n = 3). (**d**) [^3^H]-2-deoxyglucose uptake was assessed at baseline and in response to 100 nM insulin and 10 nM IGF1. Data are expressed relative to basal control values (n = 4). (**e)** Basal extracellular acidification rate (ECAR) in control and IR-Mut iPSC, expressed relative to control and normalised by DNA concentration (n = 3). (**f**) Lactate concentrations in conditioned medium at 0 and 4 hours after addition of fresh media and normalised to cellular protein content (n = 4). *indicates p < 0.05 for IR-Mut vs. control.

To specifically examine genes associated with glucose metabolism that may be modulated along with the impaired oxidative metabolism, microarray data were queried for significant changes in the KEGG glycolysis/gluconeogenesis gene set (p < 0.05; Fig. 4a, Supplementary Table S3). Notably, expression of several genes regulating glucose metabolism, including hexokinase 1 (*HK1*), aldolase C (*ALDOC*), phosphoglucomutase 1 (*PGM1*), and pyruvate kinase, muscle isozyme 2 (*PKM2*), were significantly decreased in IR-Mut iPSC (Fig. 4b). qRT-PCR revealed reduced mRNA expression of the glucose transporters *GLUT1* (*SLC2A1*) and *GLUT4* (*SLC2A4*) and glycolytic enzymes *HK1*, *ALDOA*, and *PKM2* in IR-Mut iPSC (Fig. 4c). Protein expression of hypoxia-inducible factor 1 alpha (HIF1α), a transcription factor subunit that responds to hypoxia and energetic stress and promotes glycolytic compensation, was undetectable in both the control and IR-Mut iPSC (not shown).

Given these patterns suggesting that glycolytic metabolism could be altered in insulin resistant iPS cells, we next determined cellular glucose uptake using radiolabelled 2-deoxyglucose (2-DG). Under basal conditions, control and IR-Mut iPSC had similar 2-DG uptake (Fig. 4d, left bars). Upon stimulation with 100 nM insulin or 10 nM IGF1, both control and IR-Mut iPSC showed small increases in 2-DG uptake, which were not significantly different from each other (Fig. 4d, middle and right bars). Anaerobic glucose metabolism was measured by both extracellular acidification rate (ECAR; Seahorse Bioanalyzer) and lactate release into conditioned media. There was no difference in basal ECAR (measured after 60-min incubation in 5 mM glucose in 5-min intervals) between control and IR-Mut iPSC (Fig. 4e). However, with prolonged incubation (4 hrs), insulin-resistant iPSC had an 82% increase in lactate levels in conditioned media (p = 0.002; Fig. 4f), despite unaltered activity of lactate dehydrogenase (not shown). These data suggest that IR-Mut iPSC have increased reliance on glycolytic metabolism.

The above data indicate that multiple metabolic pathways are impaired with insulin resistance, potentially due to coordinated transcriptional regulation. Promoter analysis of all significantly downregulated genes highlights enrichment for gene promoters containing transcription factor Sp1 (SP1), nuclear factor of activated T-cells, cytoplasmic (NFATC), and jun proto-oncogene (JUN) motifs in IR-Mut iPSC (Supplementary Table S4). In addition, expression of *NFATC1* (not shown) and *JUN*^25^ mRNA were also downregulated in the IR-Mut iPSC. *SP1* mRNA was significantly increased by more than 3-fold in IR-Mut iPSC (p = 0.02), while transcriptional modifier YY1 (also known as Ying Yang 1), a PGC1α target, was decreased (p = 0.02; Supplementary Fig. S4B). Furthermore, expression of several tumour protein p53-related genes, which regulate both cell proliferation and metabolism^26^, was significantly altered in the IR-Mut iPSC (Supplementary Table S5), including decreased expression of p63, p73, and p53-inducible ribonucleotide reductase and increased expression of p53 and p53-binding protein 1.

**Figure 5:**
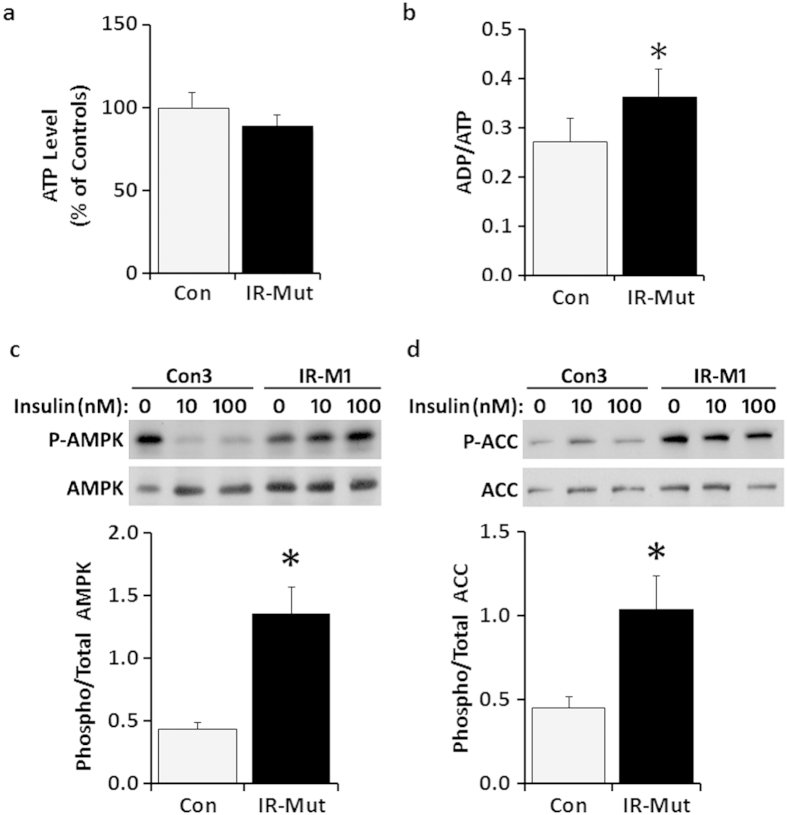
Decreased energy production in IR-Mut iPSC. (**a**) ATP level expressed relative to control (n = 3). (**b**) Ratio of ADP/ATP for control and IR-Mut iPSC (n = 3). (**c**) Representative western blot and quantification of phosphorylated to total AMPK expression ratio (n = 3). (**d**) Representative western blot and quantification of phosphorylated to total ACC expression ratio (n = 3). *p < 0.05 for IR-Mut vs. control.

To ascertain the net impact of decreased aerobic metabolic activity on energy production, ATP and ADP levels were measured. Insulin-resistant iPSC had a trend for decreased ATP content (19%, p = 0.07; Fig. 5a) and a significant 34% increase in ADP/ATP ratio (p = 0.04; Fig. 5b), consistent with inefficient production or maintenance of ATP levels. This energy deficit was not associated with decreased viability (Supplementary Fig. S5A). Reduced energy production can lead to phosphorylation and activation of AMP-activated protein kinase (AMPK), a key monitor of cellular energy status. Indeed, insulin-resistant iPSC showed a 3-fold increase in phosphorylated AMPK (p < 0.001) and a 2-fold increase in the phosphorylation of its downstream target acetyl-CoA carboxylase (ACC; p < 0.001), with no change in total protein levels (Fig. 5c,d).

**Figure 6:**
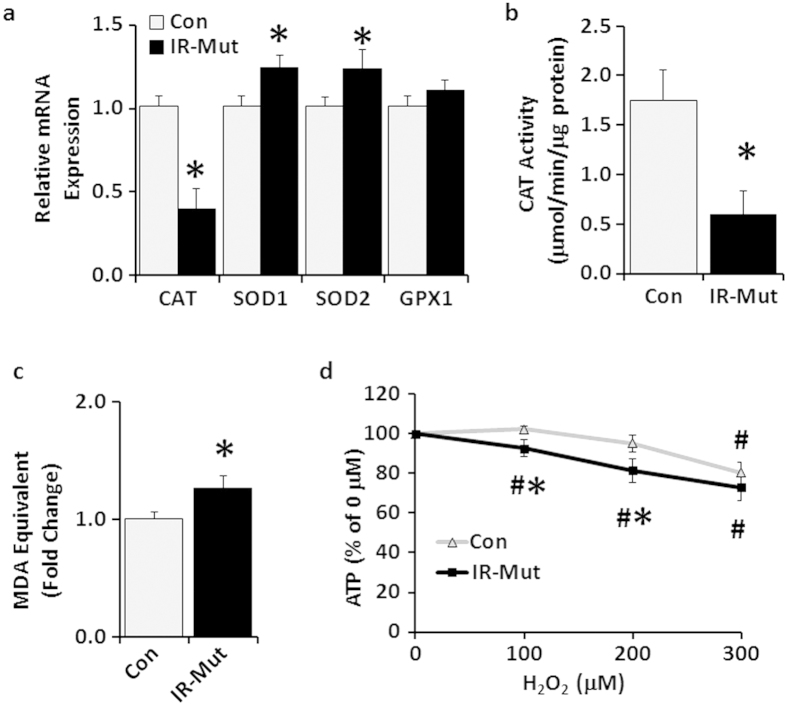
IR-Mut iPSC have increased oxidative stress. (**a**) Relative mRNA expression of antioxidant enzymes, as assessed by qRT-PCR. Data are normalised to *36B4* and expressed relative to control (n = 3). (**b**) Catalase activity was measured as μmole/min and normalised to cellular protein (n = 3). (**c**) Lipid peroxidation was calculated by MDA equivalents using TBARS assay (n = 3). (**d**) ATP level expressed relative to vehicle (0 μM H_2_O_2_) (n = 2 of 4 Con vs 4 IR-Mut and n = 1 of 2 Con vs 3 IR-Mut). *p < 0.05 for IR-Mut vs. control. ^#^p < 0.05 vs. vehicle.

Dysregulation of mitochondrial function can lead to increased reactive oxygen species (ROS) production, which in turn could be linked to insulin resistance^27^. Therefore, we examined expression of genes regulating oxidative stress. Insulin-resistant iPSC had modest, but significant, ~20% increases in *SOD1* and *SOD2* expression (p < 0.001; Fig. 6a). By contrast, there was a robust 50% decrease in catalase (*CAT*) expression (p < 0.001; Fig. 6a), with a coordinate 66% decrease in CAT activity (p = 0.01; Fig. 6b). There was a trend for increased protein oxidation (assessed by OxyBlot; Supplementary Fig. S5B,C), and lipid peroxidation (TBARS assay) was increased 26% in insulin-resistant IR-Mut iPSC (p = 0.03; Fig. 6c).

To determine whether insulin resistance increased the susceptibility of iPSC to oxidative stress, cells were treated with hydrogen peroxide for 1 hour. Control cells displayed no significant reduction in ATP levels at the lower doses of hydrogen peroxide tested (100 and 200 μM). By contrast, ATP levels were decreased in IR-Mut iPSC by 7 and 18% in response to 100 and 200 μM H_2_O_2_, respectively, as compared with vehicle (p < 0.01; Fig. 6d). These ATP values were also significantly lower than control iPSC levels (p < 0.05). At the highest concentration of H_2_O_2_ tested, ATP levels were significantly decreased in both control and IR-Mut cells by 20–27% compared to vehicle, with no difference between groups.

## Discussion

Insulin resistance is an important predictor of diabetes risk^1^ and is associated with impairments in energy metabolism in both mice and humans. *In vivo*, it is difficult to discern whether the insulin resistance or the oxidative dysfunction is the primary event, given the close interrelationships between these phenotypes. iPS cells are a robust tool for studying human disease pathology. This *in vitro* human model system can be used for in depth molecular, biochemical, and developmental studies of complex diseases. iPS cells also allow identification of cell autonomous phenotypes present in the absence of differentiation-dependent confounders. We demonstrate that stem cells with primary insulin resistance exhibit profound impairments in cellular energy metabolism that result from the collective impact of several metabolic defects.

Firstly, IR-Mut iPS cells have alterations in mitochondrial size and number indicative of increased mitochondrial fission. This is supported by finding of increased expression of MFF and INF2, both of which promote mitochondrial fission. However, total mitochondrial mass, DNA content, and citrate synthase protein content are similar between the insulin resistant cells and controls. Such differences in mitochondrial number, despite similar mass, also are consistent with studies showing the ability of insulin signalling to promote mitochondrial fusion^28^,^29^,^30^,^31^. Enhanced mitochondrial fission has also been observed in terminally differentiated tissues from patients with type 2 diabetes, including skeletal muscle and pancreatic beta cells^29^,^32^, and our current data suggest that such changes may be programmed even in the pre-differentiation state. These data are particularly interesting as smaller mitochondria produce ATP less efficiently due to decreased expression of mtDNA-encoded subunits and mitochondrial content mixing^33^,^34^,^35^ and, thus, could contribute to reduced oxidative capacity in IR-Mut iPSC or differentiated tissues such as skeletal muscle^36^,^37^.

Secondly, IR-Mut iPS cells demonstrate distinct phenotypes indicating dysregulation of oxidative metabolism. Lactate levels in conditioned media are strikingly increased in insulin resistant iPSC, and this pattern was also observed in mesenchymal progenitor cells (MPC) derived from these insulin resistant iPS cells, indicating this is a phenotype that can be identified in at least two distinct populations of progenitor cells^38^. In parallel, endogenous citrate synthase enzymatic activity was markedly reduced in IR-Mut cells. Again, these findings are similar to the increased plasma lactate levels^39^,^40^,^41^, and decreased citrate synthase activity in skeletal muscle^37^,^42^ and adipose tissue^43^ with insulin resistance and T2D. In the iPS cells, differences in citrate synthase activity resolved after addition of exogenous oxaloacetate, indicating that substrate availability might be limiting for TCA cycle metabolism in insulin resistance. Whether the same is true *in vivo* still needs to be resolved^44^,^45^,^46^. Similarly, our analysis revealed that spare respiratory capacity was reduced by more than 50% in insulin resistant cells.

Collectively, these results may indicate that primary insulin resistance can result in impaired oxidative capacity, with a shift in balance toward nonoxidative metabolism. Despite this shift, insulin resistant cells are incapable of increasing energy production, and thus unable to adapt to conditions promoting energy deficit or increased energetic stress, such as exercise. These data are also consistent with the decreased OXPHOS activity and ATP production in animals with insulin receptor mutations^47^ and with recent data from humans with insulin receptor mutations demonstrating decreases in phosphocreatine recovery after exercise^8^.

Though stem cells have functional OXPHOS complexes and mitochondrial networks, they largely depend upon glycolysis for ATP production^48^,^49^,^50^,^51^, and a shift to glycolysis is necessary for reprogramming and establishment of pluripotency^52^,^53^. The observed increase in lactate production in IR-Mut iPSC may indicate an even greater degree of dependence on glycolytic metabolism in these cells, potentially in response to their defective oxidative metabolism. However, the increased ADP/ATP ratio and AMPK activation in IR-Mut cells indicate that glycolytic energy metabolism is insufficient to compensate, resulting in inability to maintain normal energy stores. This inadequate energy availability may also contribute to decreased proliferation (as we have previously reported^25^), thus impairing an essential component of stem cell function. In turn, such reductions in proliferation of stem cells and other developmentally important progenitors could also potentially play a role in the diminished prenatal growth in patients with Donohue syndrome.

Imbalances between ROS production and antioxidant responses are linked to insulin resistance in both rodents and humans^27^,^54^,^55^. Experimental increases in ROS and induction of mitochondrial dysfunction can provoke insulin resistance^47^,^56^, and treatment with antioxidants can alleviate insulin resistance^27^. Here, we demonstrate that both mitochondrial dysfunction and increased oxidative stress develop in cells that have primary, i.e. genetically-determined, insulin resistance. Insulin resistant iPSC exhibit increased sensitivity to hydrogen peroxide. These defects may be linked, in part, to the profound reduction in expression and activity of the antioxidant enzyme catalase. Interestingly, catalase mRNA was also significantly reduced in MPCs derived from these IR-Mut iPSC^38^. Since increased expression of catalase in mitochondria prevents aging-induced mitochondrial dysfunction, as well as lipid-induced insulin resistance^57^, it is possible that insulin resistance itself may both impair mitochondrial function and ability to clear ROS, thus promoting increased oxidative stress and initiating a vicious cycle of metabolic defects.

We do not yet know the specific transcriptional regulatory molecules responsible for the altered metabolism in insulin resistance. Transcriptome and promoter analysis suggests a potential role for genes regulated by PGC1α, SP1, NFATC, JUN, and p53. p53 is a possible regulator as it controls not only cell proliferation^25^, but also reduces transcription of key metabolic regulatory genes including glucose transporters and glycolytic genes hexokinases 1 and 2, 6-phosphofructo-2-kinase/fructose-2,6-biphosphatase 3 (*PFKFB3*), phosphofructokinase isozymes M and P, and phosphoglucomutase, while increasing glutaminase 2 expression^58^,^59^,^60^. Consistent with this possibility, expression of glucose transporters 1 and 4, *HK1*, and *PGM1* was decreased in IR-Mut iPSC. However, glutaminase 2, which plays an important role in oxidative stress response, was also downregulated in IR-Mut cells. Moreover, mRNA expression of the coactivator PGC1α and its target YY1 were also downregulated in IR-Mut cells – a pattern similar to the decreased PGC1α expression in muscle, adipose, and liver of humans with insulin resistance and in INSR-knockdown C2C12 myotubes^18^,^61^,^62^. Since both PGC1α and YY1 are key regulators of mitochondrial metabolism, and responsive to cellular growth and nutrient status via interactions with mechanistic target of rapamycin (serine/threonine kinase) (mTOR)^63^,^64^,^65^, perturbations in these pathways could contribute to observed phenotypes. We did not observe changes in MTOR-dependent activity (phosphorylation of S6 ribosomal protein) or p53 protein or acetylation in IR-Mut cells (data not shown), indicating that insulin receptor mutations do not impair all regulatory pathways.

Our study in iPS cells demonstrates an important role for insulin resistance as a driver of mitochondrial oxidative dysfunction, even prior to differentiation of cells into more classical insulin target tissue cell types, such as muscle, fat and liver. At present, our study is limited to severe genetic mutations in the insulin receptor as the cause for insulin resistance, and some between-patient differences may be related to the specific insulin receptor mutation, the genetic background of the individual, or residual epigenetic differences maintained during cellular reprogramming^66^,^67^. Nevertheless, these data support the importance of future studies of cells from individuals with more common and less severe forms of insulin resistance, such as patients with T2D. Comparative studies in these additional cell lines would be beneficial in determining the primary and fundamental metabolic phenotypes associated with insulin resistance in stem cell populations and their influence on developmental trajectories.

In summary, our results demonstrate that primary insulin resistance can lead to reduced mitochondrial oxidative function, increased reliance on anaerobic metabolism, and impaired ability to respond to conditions of maximal respiratory demand. Collectively, these defects contribute to not only impaired energy homeostasis, but also increase oxidative stress. Moreover, these data highlight that phenotypes associated with human insulin resistance can be recapitulated in a cell culture system, allowing future analyses of mechanisms by which defects in insulin signalling and cellular metabolism alter the function of key stem cell populations.

## Methods

Generation and use of human iPSC was approved by Joslin Diabetes Center’s Committee on Human Studies. All methods were performed in accordance with the approved guidelines. The pre-existing human fibroblast lines used to generate iPSC are available from commercial sources, with no identifiable private information provided to the study investigators.

### Reagents

Primary antibodies included anti-citrate synthase (abcam ab96600), anti-fumarase (Novus Biologicals NBP1-31336), MitoProfile Total OXPHOS Cocktail (abcam ab110413), anti-phospho-AMPKalpha (Thr172; Cell Signaling 2535), anti-AMPKalpha (Cell Signaling 2532), anti-phospho-acetyl-CoA carboxylase (Cell Signaling 3661), anti-acetyl-CoA carboxylase (Cell Signaling 3662), and anti-VDAC (Cell Signaling 4866).

### iPSC Culture

iPSC were generated as previously described^25^ and grown on Geltrex-coated plates in mTesR1 media (StemCell Technologies). Cells were studied at similar passage numbers and at approximately 75% confluency.

### Western Blot Analysis

iPSC were lysed in 1% sodium dodecyl sulfate, and protein concentrations were determined by BCA (Thermo Scientific). Equal amounts of protein were separated by SDS-PAGE and transferred to nitrocellulose membranes, blocked with 5% nonfat dry milk in Tris-buffered saline with 0.1% Tween, incubated with primary antibodies overnight, and washed prior to incubation with HRP-conjugated secondary antibodies. Proteins were detected by enhanced chemiluminescence (PerkinElmer Life Sciences). For protein oxidation analysis, samples were processed using an OxyBlot protein oxidation detection kit (Millipore). For quantitation, band intensities were measured using Adobe Photoshop on inverted scanned images; target bands were selected with rectangular marquee tool, and the average pixel intensity was determined using the histogram function.

### RNA Expression Analysis

Microarray analyses were performed as previously reported^25^. Data were analysed using the limma package from Bioconductor, with results selected by nominal p-value (p < 0.05). For qRT-PCR analysis, RNA was isolated using RiboZol (AMRESCO), and 1 μg was used to synthesise cDNA (High Capacity cDNA Reverse Transcription Kit, Life Technologies). Amplification was performed using iTaq Universal SYBR Green Supermix (Bio-Rad) and ABI 7900HT Fast Real-Time PCR System (Life Technologies). The gene *36B4* was used for normalisation, and the average of the control cell values was used for calculating relative expression using the ΔΔCT method^68^. Overrepresentation of transcription factor binding sites was assessed using mSigDB (Broad Institute)^69^. Supplementary Table S6 lists the gene symbols with the coordinate gene name and primer sequences used for qRT-PCR.

### Cellular Metabolism Phenotypic Analysis

Equal numbers of cells were incubated with Kreb’s Ringer buffer (KRB; 110 nM NaCl, 4.7 nM KCl, 2 mM MgSO_4_, 1.2 mM Na_2_HPO_4_, 0.24 mM MgCl_2_) with 5 mM glucose and 0.5% bovine serum albumin (BSA) for one hour. OCR and ECAR were measured using XF24 Flux Analyzer (Seahorse Biosciences). Data were collected at baseline and at 5-minute intervals following sequential addition of oligomycin (10 μg/ml), 2,4-dinitrophenol (DNP; 10 μM) and potassium cyanide (KCN; 50 μM) and then normalised by DNA content.

### Lactate Production

Equal numbers of cells were plated per well in a 12-well plate, washed with PBS, and incubated in KRB with 5 mM glucose and 0.5% bovine serum albumin (BSA). Conditioned media were sampled at baseline and at 2 and 4 hours for measurement of lactate concentration using a colourimetric kit (Biomedical Research Service Center); values were normalised to cellular protein content.

### Enzymatic Assays

Endogenous and maximal CS activity was measured using a colourimetric assay (Sigma-Aldrich). Levels of adenine nucleotides were measured by a modified coupled enzymatic assay^70^. Cells were plated in an opaque 96-well plate. To initiate reaction, 100 μL of CellTiter-Glo luminescent assay reagent (Promega) were added to each well. After incubation for 15 minutes, intensity values were determined on a microplate reader. Next, 1.5 mM phosphoenolpyruvate (PEP) and 2.3 units/ml pyruvate kinase (PK) were added to convert ADP to ATP, and values were obtained after 2 min. Finally, 36 units/ml adenylate kinase were added to each well, and measurements were obtained after 2 min. Relative ADP levels were determined by subtracting the post-PEP/PK from basal values. Relative AMP levels were determined by subtracting the post-AK from post-PEP/PK values. Catalase activity was measured using a colourimetric kit (Biomedical Research Service Center). Lipid peroxidation was measured using a TBARS assay kit (Cayman Chemical).

### Mitochondrial DNA Content

Total DNA was prepared by lysis with 50 mM NaOH. After boiling for 5 min at 95 °C, lysates were neutralised by addition of 1 M Tris (pH 6.8). Mitochondrial DNA content was measured by qRT-PCR (Applied Biosystems 7900) using primers for *mt-ND1* and *mt-ND2*; values were normalised to the nuclear gene *ACTB*.

### Glucose Uptake

Following 4-hour serum starvation in KRB buffer with 0.5% BSA, cells were treated with PBS, 100 nM insulin, or 10 nM insulin-like growth factor 1 (IGF1) for 30 minutes, and then incubated with^3^H 2-deoxyglucose (1:20 in cold 5.1 mM 2-deoxyglucose) for 10 minutes. Cells were lysed in 1% Triton X-100, and radioactivity was determined by scintillation counting. Values were normalised to protein content (BCA).

### Transmission Electron Microscopy

iPSC in suspension were fixed in 2.5% glutaraldehyde, 0.1 M phosphate buffer (pH 7.4) for 1 hour before several washes in phosphate buffer and 1-hr post-fixation in 2% OsO4 (in phosphate buffer). After ethanol dehydration (50/70/95/100%) and clearing with propylene oxide, propylene oxide and Araldite 502 epoxy resin (1:1) with vacuum desiccation were used for resin infiltration. Pellets were embedded and cured in fresh resin using BEEM capsules. Sections (80 nM) stained with uranyl acetate and lead citrate were imaged on 75-mesh copper grids with a Philips 301 transmission electron microscope. Micrographs were taken for quantification of mitochondrial number (4500X) and area (34000X) using ImageJ the cell counter function to count mitochondria and the freehand selection to measure cellular and mitochondrial areas.

### Statistical Analysis

Data are presented as mean ± SEM. Significant differences were analysed by Student’s t-test (p values), with p < 0.05 considered significant.

## Additional Information

**How to cite this article**: Burkart, A. M. *et al.* Insulin Resistance in Human iPS Cells Reduces Mitochondrial Size and Function. *Sci. Rep.* **6**, 22788; doi: 10.1038/srep22788 (2016).

## Supplementary Material

Supplementary Information
