# Supplementary material for: Insulin Resistance in Human iPS Cells Reduces Mitochondrial Size and Function
Source: Sci Rep. 2016 Mar 7;6:22788. doi: 10.1038/srep22788 (PMC4780029; doi:10.1038/srep22788)
Supplement: Supplementary Information [file srep22788-s1.pdf]

Insulin Resistance in Human iPS Cells Reduces Mitochondrial Size and Function

Alison M. Burkart, Kelly Tan, Laura Warren, Salvatore Iovino, Katelyn J. Hughes, C. Ronald Kahn, and Mary-Elizabeth Patti*

Integrative Physiology and Metabolism Research Division, Joslin Diabetes Center and Harvard Medical School, Boston, MA, 02215, USA.

*Correspondence:
Mary-Elizabeth Patti MD
1 Joslin Place, Boston, MA 02215
Phone: 617-309-1966
FAX: 617-309-2593
Mary.Elizabeth.Patti@joslin.harvard.edu

Supplemental Information: 5 Figures; 6 Tables

Supplementary Figures

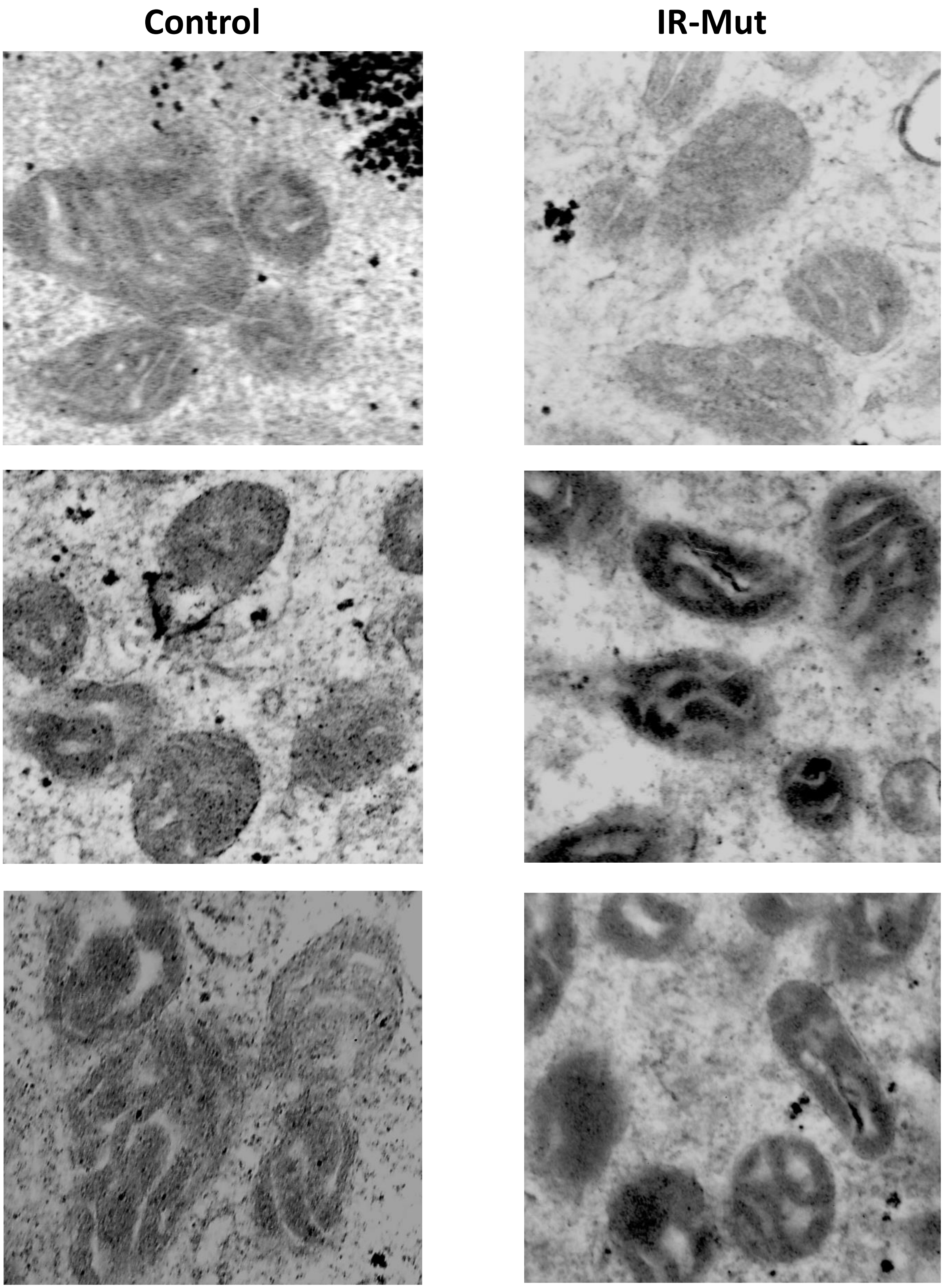

Supplementary Figure S1: IR-Mut iPSC have smaller mitochondria.
Representative electron micrographs of control and IR-Mut iPSC (34000x).

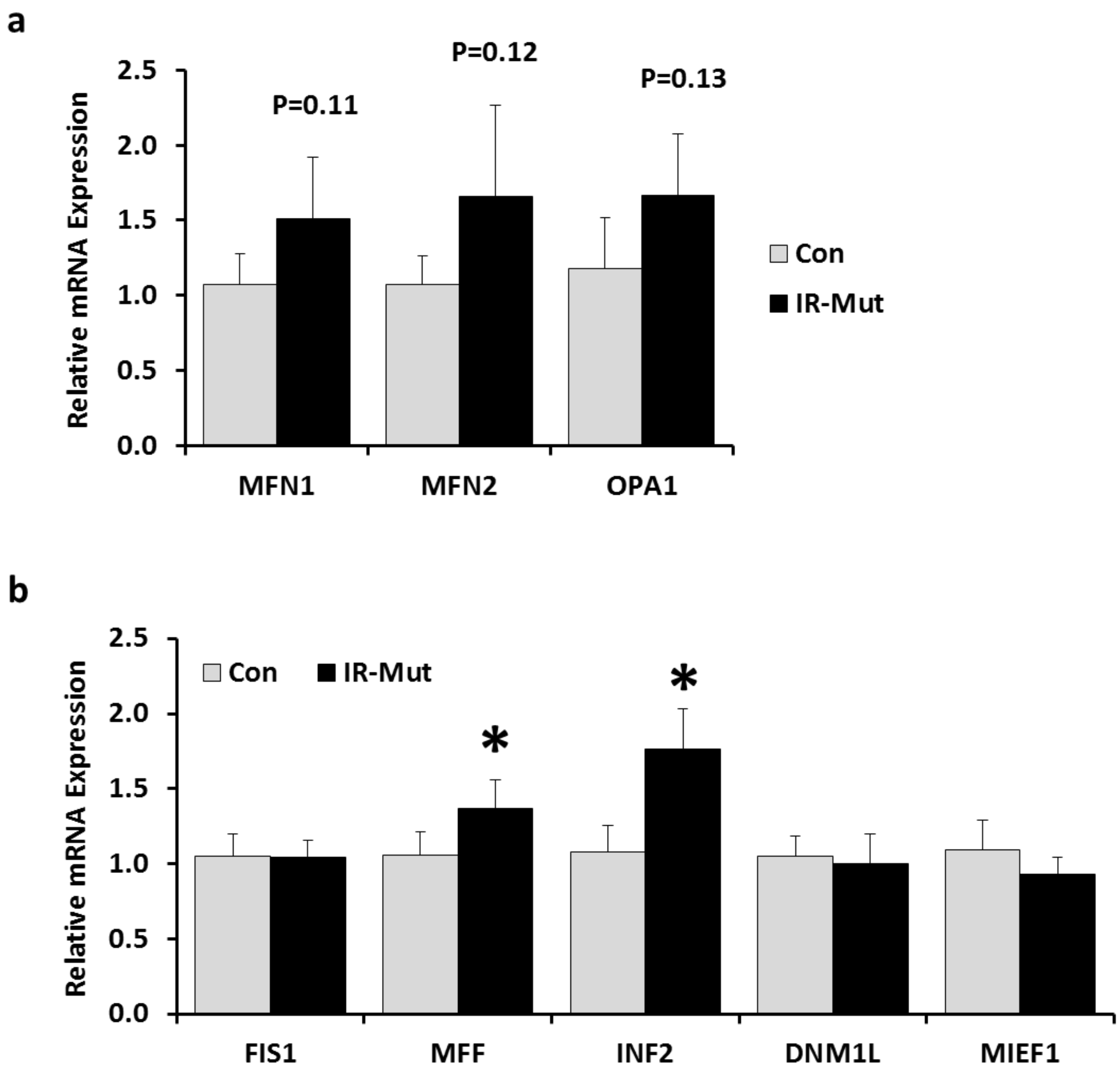

Supplementary Figure S2: Genes regulating mitochondrial fission show increased expression in IR-Mut iPSC.
Relative mRNA expression of proteins regulating mitochondrial fusion (A) and fission (B) dynamics, as assessed by qRT-PCR. Data are normalised to *36B4* and expressed relative to control (n=3). * $p<0.05$ for IR-Mut vs. control.

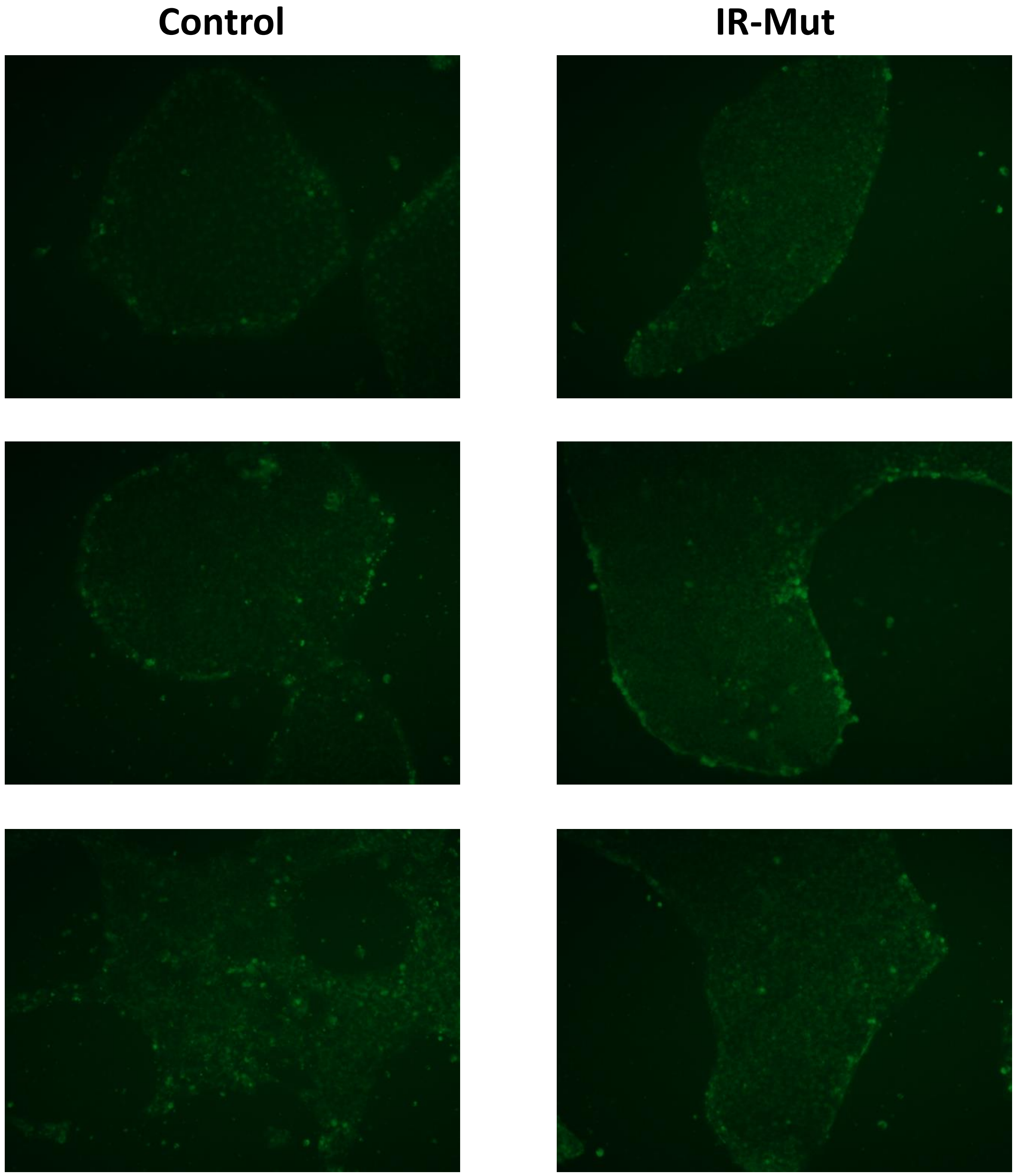

Supplementary Figure S3: IR-Mut iPSC have similar mitochondrial mass to control iPSC. MitoTracker Green staining of control and IR-Mut iPS cells. Representative images are shown.

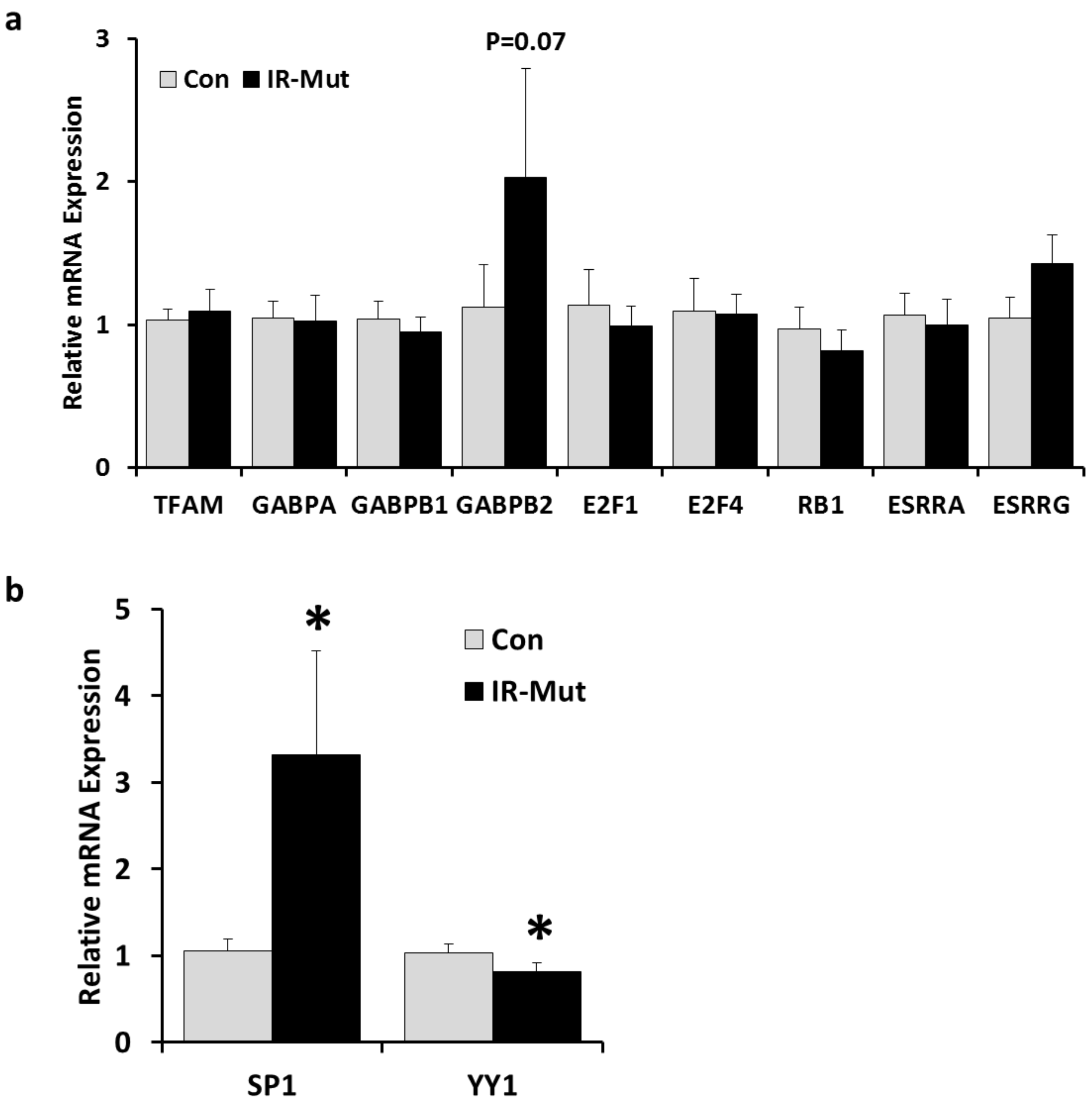

Supplementary Figure S4: Transcriptional regulators SP1 and YY1 are altered in IR-Mut iPSC.
A. Relative mRNA expression of transcriptional regulators of metabolism, as assessed by qRT-PCR. B. Relative mRNA expression of SP1 and YY1, as assessed by qRT-PCR. Data are normalised to *36B4* and expressed relative to control (n=3). * $p<0.05$ for IR-Mut vs. control

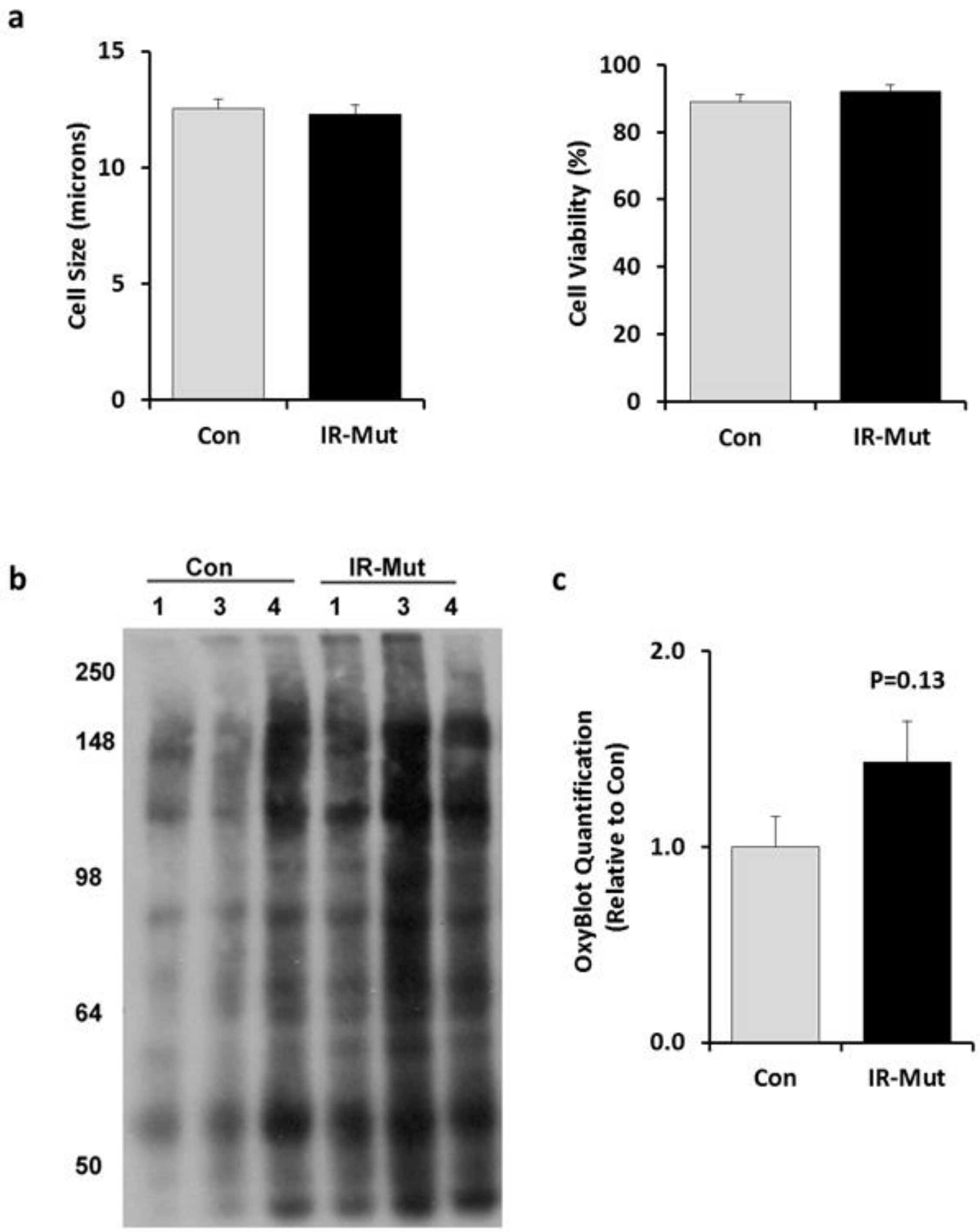

Supplementary Figure S5: Tendency for increased protein oxidation in IR-Mut iPSC.

A. Cell size (in microns) and viability based on trypan blue exclusion using Cellometer. B. Representative image of OxyBlot analysis. C. Quantification of OxyBlot analysis, expressed relative to control iPSC values.

Supplementary Tables

Supplementary Table S1: Clinical information for subjects, including sex, age, genotype, and clinical phenotype, with a summary of the signalling defects observed previously[25].

		Sex	Age	Genotype	Phenotype	Signaling Defect
**Con1**	**BJ**	M	Newborn	Normal	Healthy	None
**Con2**	**GM05400**	M	6 years	Normal	Healthy	None
**Con3**	**GM00409**	M	7 years	Normal	Healthy	None
**Con4**	**GM00969**	F	2 years	Normal	Healthy	None
**Con5**	**GM00038**	F	9 years	Normal	Healthy	None
**IR-Mut1**	**Minn1**	F	1 month	Exon 14 (nonsense; A897X)	Low birth weight; Insulin resistance; Characteristic facial appearance; No subcutaneous adipose tissue	INSR expression <10%, INSR activation <50% IGF1R activation 100%
**IR-Mut2**	**GM10277**	F	15 years	Exon 1 (missense; A2G)	Pre- and post-natal growth retardation; Microcephaly; Mental retardation; Pachydermy; Severe insulin resistance	INSR expression 100%, INSR activation <20% IGF1R activation 100%
**IR-Mut3**	**GM20034**	M	2 months	Exon 3 (missense; L233P)	Intrauterine growth restriction; no subcutaneous adipose tissue; failure to thrive	INSR expression <5%, INSR activation <5% IGF1R activation 100%
**IR-Mut4**	**GM20327**	F	2 months	Exon 2 (nonsense; E124X)	Severe intrauterine growth restriction; dysmorphic features; poor weight gain after birth; hyperglycaemia; insulin resistance	INSR expression <5%, INSR activation <50% IGF1R activation 100%

Supplementary Table S2: Significantly altered genes (nominal p<0.05) associated with mitochondria based on gene ontology using the term "mitoc*".

ID	Gene Symbol	Gene Title	Log2 FC	P-Value
11716302_s_at	ACSL1	acyl-CoA synthetase long-chain family member 1	-0.75	0.000
11758498_at	DNM3	dynamin 3	-1.35	0.001
11724723_a_at	HSPA2	heat shock 70kDa protein 2	-0.96	0.001
11732524_a_at	CHKB-CPT1B /// CPT1B	choline kinase-like, carnitine palmitoyltransferase 1B (muscle) transcription unit /// carnitine palmitoyltransferase 1B (muscle)	0.97	0.001
11733255_a_at	CHKB-CPT1B /// CPT1B	choline kinase-like, carnitine palmitoyltransferase 1B (muscle) transcription unit /// carnitine palmitoyltransferase 1B (muscle)	0.86	0.001
11716824_s_at	BDH2	3-hydroxybutyrate dehydrogenase, type 2	0.43	0.002
11755448_s_at	CAPRIN2	caprin family member 2	-0.53	0.002
11757085_s_at	ASAH2 /// ASAH2B /// ASAH2C	N-acylsphingosine amidohydrolase (non-lysosomal ceramidase) 2 /// N-acylsphingosine amidohydrolase (non-lysosomal ceramidase) 2B /// N-acylsphingosine amidohydrolase (non-lysosomal ceramidase) 2C	0.44	0.002
11749782_x_at	BCAT1	branched chain amino-acid transaminase 1, cytosolic	0.46	0.003
11722231_at	PDP1	pyruvate dehyrogenase phosphatase catalytic subunit 1	-0.90	0.003
11761711_at	FEZ1	fasciculation and elongation protein zeta 1 (zygin I)	0.34	0.003
11721935_at	DNAJC27	DnaJ (Hsp40) homolog, subfamily C, member 27	0.39	0.003
11732360_a_at	CTPS2	CTP synthase II	0.69	0.003
11760037_a_at	GLS	glutaminase	-0.42	0.004
11750700_a_at	ACSL1	acyl-CoA synthetase long-chain family member 1	-0.47	0.004
11763202_x_at	FAM65B	family with sequence similarity 65, member B	-0.55	0.004
11722230_a_at	PDP1	pyruvate dehyrogenase phosphatase catalytic subunit 1	-0.71	0.004
11743104_at	PTCD3	Pentatricopeptide repeat domain 3	-0.41	0.004
11716707_a_at	ARL2BP	ADP-ribosylation factor-like 2 binding protein	0.38	0.005
11756513_a_at	SFXN5	sideroflexin 5	0.44	0.005
11724062_a_at	RAD51C	RAD51 homolog C (S. cerevisiae)	0.76	0.005
11749895_a_at	BCAT1	branched chain amino-acid transaminase 1, cytosolic	0.45	0.006
11749780_a_at	BCAT1	branched chain amino-acid transaminase 1, cytosolic	0.44	0.006
11715477_at	TFRC	transferrin receptor (p90, CD71)	-0.40	0.007
11750293_a_at	RAD51C	RAD51 homolog C (S. cerevisiae)	0.90	0.007
11732520_a_at	RAD51C	RAD51 homolog C (S. cerevisiae)	0.86	0.007
11727797_at	NDFIP2	Nedd4 family interacting protein 2	-0.39	0.007
11762275_x_at	UQCRB	ubiquinol-cytochrome c reductase binding protein	0.48	0.008
11761116_a_at	FAM65B	family with sequence similarity 65, member B	-0.53	0.008
11732575_a_at	FAM65B	family with sequence similarity 65, member B	-0.56	0.008
11721707_at	GPAM	glycerol-3-phosphate acyltransferase, mitochondrial	0.27	0.008
11751154_x_at	ALDOC	aldolase C, fructose-bisphosphate	-0.47	0.008
11717297_at	LRPPRC	leucine-rich PPR-motif containing	-0.64	0.009
11751680_a_at	UQCRB	ubiquinol-cytochrome c reductase binding protein	0.52	0.009
11723251_at	RPS15A	ribosomal protein S15a	0.42	0.009
11718765_a_at	NDUFA6	NADH dehydrogenase (ubiquinone) 1 alpha subcomplex, 6, 14kDa	0.26	0.010
11751064_a_at	EXOG	endo/exonuclease (5'-3'), endonuclease G-like	0.40	0.010
11752103_a_at	HK1	hexokinase 1	-0.49	0.010
11721736_s_at	TNFRSF19	tumor necrosis factor receptor superfamily, member 19	1.58	0.010

11723105_at	CMPK2	cytidine monophosphate (UMP-CMP) kinase 2, mitochondrial	0.38	0.010
11725634_at	MRPS25	mitochondrial ribosomal protein S25	0.25	0.010
11756156_s_at	TFRC	transferrin receptor (p90, CD71)	-0.39	0.011
11718291_a_at	HK1	hexokinase 1	-0.47	0.011
11763693_a_at	GLS	glutaminase	-0.28	0.011
11747314_a_at	PDP1	pyruvate dehyrogenase phosphatase catalytic subunit 1	-0.59	0.011
11751442_s_at	SLC25A44	solute carrier family 25, member 44	0.33	0.011
11718213_a_at	SLC27A2	solute carrier family 27 (fatty acid transporter), member 2	0.81	0.011
11730958_a_at	CHCHD4	coiled-coil-helix-coiled-coil-helix domain containing 4	0.32	0.011
11750890_a_at	MARS2	methionyl-tRNA synthetase 2, mitochondrial	-0.26	0.011
11749287_a_at	ARL2BP	ADP-ribosylation factor-like 2 binding protein	0.38	0.011
11757825_a_at	C7orf55 /// LUC7L2	chromosome 7 open reading frame 55 /// LUC7-like 2 (S. cerevisiae)	0.34	0.012
11727954_at	PACS2	phosphofurin acidic cluster sorting protein 2	0.35	0.012
11755807_a_at	DNA2	DNA replication helicase 2 homolog (yeast)	0.27	0.013
11731491_a_at	HK1	hexokinase 1	-0.46	0.013
11763993_s_at	ATPAF1	ATP synthase mitochondrial F1 complex assembly factor 1	0.25	0.013
11750043_a_at	MTG1	mitochondrial GTPase 1 homolog (S. cerevisiae)	0.33	0.013
11749098_a_at	CAPRIN2	caprin family member 2	-0.47	0.014
11736211_at	MRP63	mitochondrial ribosomal protein 63	0.49	0.014
11726334_s_at	CRLS1	cardiolipin synthase 1	0.39	0.014
11757304_s_at	ARL2BP	ADP-ribosylation factor-like 2 binding protein	0.32	0.014
11747079_x_at	ALDOC	aldolase C, fructose-bisphosphate	-0.43	0.014
11730021_s_at	ACSM2A /// ACSM2B	acyl-CoA synthetase medium-chain family member 2A /// acyl-CoA synthetase medium-chain family member 2B	0.29	0.014
11739877_x_at	BSG	basigin (Ok blood group)	-0.50	0.014
11720180_at	DDX28	DEAD (Asp-Glu-Ala-Asp) box polypeptide 28	0.32	0.015
11756286_a_at	SFXN2	sideroflexin 2	1.00	0.015
11728686_a_at	SFXN1	sideroflexin 1	0.43	0.015
11739601_at	AASS	aminoadipate-semialdehyde synthase	-0.42	0.015
11721706_at	GPAM	glycerol-3-phosphate acyltransferase, mitochondrial	0.30	0.015
11744488_a_at	ACOT9	acyl-CoA thioesterase 9	-0.38	0.015
11763975_a_at	MRPS11	mitochondrial ribosomal protein S11	0.30	0.016
11763320_x_at	GLS	glutaminase	-0.26	0.016
11739215_a_at	SLC25A23	solute carrier family 25 (mitochondrial carrier; phosphate carrier), member 23	-0.24	0.016
11764001_x_at	MRPS11	mitochondrial ribosomal protein S11	0.27	0.016
11718292_s_at	HK1	hexokinase 1	-0.44	0.016
11728687_at	SFXN1	sideroflexin 1	0.34	0.016
11724436_a_at	GABPB1	GA binding protein transcription factor, beta subunit 1	0.32	0.017
11723613_s_at	SERHL /// SERHL2	serine hydrolase-like /// serine hydrolase-like 2	0.55	0.017
11763201_at	FAM65B	family with sequence similarity 65, member B	-0.45	0.017
11716047_a_at	C21orf33	chromosome 21 open reading frame 33	0.30	0.017
11730738_a_at	SCCPDH	saccharopine dehydrogenase (putative)	0.43	0.017
11722429_s_at	PPL	periplakin	-0.50	0.017
11741521_a_at	HAO2	hydroxyacid oxidase 2 (long chain)	0.27	0.018

11716757_a_at	DHX30	DEAH (Asp-Glu-Ala-His) box polypeptide 30	0.30	0.018
11749781_s_at	BCAT1	branched chain amino-acid transaminase 1, cytosolic	0.35	0.018
11741670_x_at	ESR2	estrogen receptor 2 (ER beta)	-0.29	0.018
11743811_a_at	TFAM	transcription factor A, mitochondrial	0.33	0.018
11760038_a_at	GLS	glutaminase	-0.26	0.018
11751153_a_at	ALDOC	aldolase C, fructose-bisphosphate	-0.35	0.018
11727324_at	NAGS	N-acetylglutamate synthase	-0.42	0.019
11729710_a_at	MARS2	methionyl-tRNA synthetase 2, mitochondrial	-0.26	0.019
11758535_s_at	GPAM	glycerol-3-phosphate acyltransferase, mitochondrial	0.28	0.019
11751388_a_at	FEN1	flap structure-specific endonuclease 1	0.27	0.019
11755241_a_at	AASS	aminoadipate-semialdehyde synthase	-0.50	0.019
11730156_a_at	WWOX	WW domain containing oxidoreductase	0.80	0.019
11758921_x_at	MRPS17	mitochondrial ribosomal protein S17	0.25	0.019
11741148_a_at	PRDX2	peroxiredoxin 2	0.37	0.020
11715734_a_at	NIPSNAP1	nipsnap homolog 1 (C. elegans)	0.38	0.020
11724057_a_at	AADAT	aminoadipate aminotransferase	0.42	0.020
11751676_x_at	AK2	adenylate kinase 2	0.34	0.021
11720140_at	RILP	Rab interacting lysosomal protein	-0.36	0.021
11724462_x_at	ALDOC	aldolase C, fructose-bisphosphate	-0.43	0.021
11717498_a_at	SLC25A36	solute carrier family 25, member 36	-0.22	0.022
11718571_at	NT5DC3	5'-nucleotidase domain containing 3	-0.38	0.022
11741402_a_at	C21orf33	chromosome 21 open reading frame 33	0.35	0.022
11739911_at	PCBD2	pterin-4 alpha-carbinolamine dehydratase/dimerization cofactor of hepatocyte nuclear factor 1 alpha (TCF1) 2	0.42	0.022
11759535_at	MRPS12	mitochondrial ribosomal protein S12	0.30	0.022
11736193_a_at	RRM2B	ribonucleotide reductase M2 B (TP53 inducible)	-0.49	0.022
11762245_a_at	RMND1	required for meiotic nuclear division 1 homolog (S. cerevisiae)	0.39	0.023
11758157_s_at	COQ10B	coenzyme Q10 homolog B (S. cerevisiae)	-0.28	0.023
11736192_at	RRM2B	ribonucleotide reductase M2 B (TP53 inducible)	-0.41	0.023
11745216_s_at	KBTBD4 /// PTPMT1	kelch repeat and BTB (POZ) domain containing 4 /// protein tyrosine phosphatase, mitochondrial 1	0.33	0.023
11751732_x_at	FAM136A	family with sequence similarity 136, member A	0.43	0.023
11746069_a_at	GATM	glycine amidinotransferase (L-arginine:glycine amidinotransferase)	-0.59	0.023
11750960_a_at	AASS	aminoadipate-semialdehyde synthase	-0.48	0.024
11760397_at	SARS2	seryl-tRNA synthetase 2, mitochondrial	0.22	0.024
11730931_at	ACSS3	acyl-CoA synthetase short-chain family member 3	0.34	0.024
11729539_a_at	IMMP1L	IMP1 inner mitochondrial membrane peptidase-like (S. cerevisiae)	0.25	0.025
11724461_a_at	ALDOC	aldolase C, fructose-bisphosphate	-0.47	0.025
11746563_x_at	FAM65A	family with sequence similarity 65, member A	-0.57	0.025
11756498_a_at	GLS2	glutaminase 2 (liver, mitochondrial)	-0.42	0.025
11728816_at	AARS2	alanyl-tRNA synthetase 2, mitochondrial (putative)	0.33	0.025
11759022_s_at	ELK3	ELK3, ETS-domain protein (SRF accessory protein 2)	-0.37	0.026
11720547_a_at	PDSS1	prenyl (decaprenyl) diphosphate synthase, subunit 1	0.39	0.026
11744689_x_at	ARMC10	armadillo repeat containing 10	0.68	0.027
11733969_a_at	SHMT1	serine hydroxymethyltransferase 1 (soluble)	0.39	0.027
11725721_a_at	MRM1	mitochondrial rRNA methyltransferase 1 homolog (S. cerevisiae)	0.33	0.028
11736212_x_at	MRP63	mitochondrial ribosomal protein 63	0.47	0.028

11755458_a_at	HDDC2	HD domain containing 2	0.28	0.029
11740522_a_at	TPO	thyroid peroxidase	-0.23	0.029
11727323_a_at	NAGS	N-acetylglutamate synthase	-0.30	0.029
11724783_a_at	MRPS18C	mitochondrial ribosomal protein S18C	0.28	0.029
11755090_a_at	MTG1	mitochondrial GTPase 1 homolog (S. cerevisiae)	0.29	0.029
11721934_at	DNAJC27	DnaJ (Hsp40) homolog, subfamily C, member 27	0.33	0.029
11761147_at	TYMP	thymidine phosphorylase	-0.35	0.029
11756474_a_at	CPT1A	carnitine palmitoyltransferase 1A (liver)	-0.51	0.030
11728428_a_at	CCDC136	coiled-coil domain containing 136	-0.27	0.030
11718045_s_at	RAB11B	RAB11B, member RAS oncogene family	0.27	0.030
11715771_a_at	MRPL13	mitochondrial ribosomal protein L13	0.24	0.031
11739876_s_at	BSG	basigin (Ok blood group)	-0.47	0.031
11725521_x_at	GLUL	glutamate-ammonia ligase	-0.24	0.031
11737897_a_at	ABCB7	ATP-binding cassette, sub-family B (MDR/TAP), member 7	-0.29	0.032
11717907_x_at	FAM136A	family with sequence similarity 136, member A	0.41	0.032
11744148_at	MRPL12	mitochondrial ribosomal protein L12	0.21	0.032
11758920_at	MRPS17	mitochondrial ribosomal protein S17	0.22	0.032
11719921_s_at	GM2A	GM2 ganglioside activator	-0.39	0.032
11729280_a_at	FAM175A	family with sequence similarity 175, member A	-0.41	0.032
11744999_a_at	TXNRD2	thioredoxin reductase 2	-0.92	0.032
11761271_x_at	CARS2	cysteinyl-tRNA synthetase 2, mitochondrial (putative)	0.35	0.033
11736507_s_at	FRMD6	FERM domain containing 6	-0.60	0.033
11747153_x_at	ACOT9	acyl-CoA thioesterase 9	-0.31	0.033
11734305_a_at	LACTB	lactamase, beta	-0.73	0.033
11736821_x_at	TP73	tumor protein p73	-0.24	0.034
11761243_at	CARKD	carbohydrate kinase domain containing	0.22	0.034
11738159_x_at	COX6B2	cytochrome c oxidase subunit VIb polypeptide 2 (testis)	-0.32	0.034
11727779_at	GHITM	growth hormone inducible transmembrane protein	0.32	0.034
11723466_a_at	MRPS11	mitochondrial ribosomal protein S11	0.25	0.034
11735706_a_at	BSG	basigin (Ok blood group)	-0.45	0.034
11725052_a_at	TOP1MT	topoisomerase (DNA) I, mitochondrial	-0.35	0.034
11722603_at	NEFH	neurofilament, heavy polypeptide	-0.60	0.035
11716774_a_at	RAI14	retinoic acid induced 14	-0.56	0.035
11745462_a_at	PRDX2	peroxiredoxin 2	0.28	0.035
11720329_at	RNMTL1	RNA methyltransferase like 1	0.27	0.035
11747729_s_at	SDHA /// SDHAP1 /// SDHAP2	succinate dehydrogenase complex, subunit A, flavoprotein (Fp) /// succinate dehydrogenase complex, subunit A, flavoprotein pseudogene 1 /// succinate dehydrogenase complex, subunit A, flavoprotein pseudogene 2	-0.47	0.035
11762158_at	PPA2	pyrophosphatase (inorganic) 2	-0.47	0.035
11736213_at	MRP63	mitochondrial ribosomal protein 63	0.23	0.036
11758127_s_at	CHCHD2	coiled-coil-helix-coiled-coil-helix domain containing 2	1.17	0.037
11718686_x_at	TXNRD2	thioredoxin reductase 2	-0.75	0.037
11730224_a_at	POLDIP2	polymerase (DNA-directed), delta interacting protein 2	0.31	0.038
11759049_at	ACSS3	acyl-CoA synthetase short-chain family member 3	0.31	0.038
11752949_a_at	COX6B2	cytochrome c oxidase subunit VIb polypeptide 2 (testis)	-0.27	0.038
11744708_a_at	LYRM1	LYR motif containing 1	0.23	0.038

11724303_a_at	COG8 /// PDF	component of oligomeric golgi complex 8 /// peptide deformylase (mitochondrial)	0.35	0.039
11745000_x_at	TXNRD2	thioredoxin reductase 2	-0.90	0.039
11758099_x_at	CHCHD2	coiled-coil-helix-coiled-coil-helix domain containing 2	1.13	0.039
11717264_a_at	ATPAF1	ATP synthase mitochondrial F1 complex assembly factor 1	0.21	0.039
11722469_a_at	ACSL6	acyl-CoA synthetase long-chain family member 6	-0.28	0.039
11756596_a_at	PDPR	pyruvate dehydrogenase phosphatase regulatory subunit	0.27	0.039
11720643_a_at	IVD	isovaleryl-CoA dehydrogenase	0.70	0.039
11753575_at	COQ7	coenzyme Q7 homolog, ubiquinone (yeast)	0.28	0.039
11751389_s_at	FEN1	flap structure-specific endonuclease 1	0.24	0.039
11744474_s_at	IDI1	isopentenyl-diphosphate delta isomerase 1	-0.29	0.040
11716484_at	UNC119B	unc-119 homolog B (C. elegans)	0.26	0.040
11720744_a_at	CROT	carnitine O-octanoyltransferase	-0.28	0.040
11729711_at	MARS2	methionyl-tRNA synthetase 2, mitochondrial	-0.19	0.041
11730406_a_at	PAK7	p21 protein (Cdc42/Rac)-activated kinase 7	-0.30	0.041
11717430_s_at	COX7A1	cytochrome c oxidase subunit VIIa polypeptide 1 (muscle)	-0.92	0.041
11723438_s_at	AK3	adenylate kinase 3	-0.43	0.041
11716670_s_at	RAB1B	RAB1B, member RAS oncogene family	0.21	0.041
11721387_at	LACTB	lactamase, beta	-0.70	0.041
11750128_s_at	IMMT	inner membrane protein, mitochondrial	0.26	0.042
11747631_x_at	XAF1	XIAP associated factor 1	0.23	0.042
11728248_a_at	TSFM	Ts translation elongation factor, mitochondrial	0.22	0.042
11754177_a_at	RRM2B	ribonucleotide reductase M2 B (TP53 inducible)	-0.36	0.043
11761093_at	DHRS4	Dehydrogenase/reductase (SDR family) member 4	0.23	0.043
11718044_s_at	RAB11B	RAB11B, member RAS oncogene family	0.23	0.044
11715409_a_at	PRDX2	peroxiredoxin 2	0.29	0.044
11748857_a_at	AK3	adenylate kinase 3	-0.38	0.045
11715410_x_at	PRDX2	peroxiredoxin 2	0.30	0.045
11745463_x_at	PRDX2	peroxiredoxin 2	0.30	0.045
11724381_at	FAM65B	family with sequence similarity 65, member B	-0.75	0.045
11748014_s_at	VDAC3	voltage-dependent anion channel 3	0.19	0.046
11739875_a_at	BSG	basigin (Ok blood group)	-0.40	0.046
11734655_x_at	ATP5F1	ATP synthase, H+ transporting, mitochondrial Fo complex, subunit B1	0.38	0.046
11739383_a_at	IDI1	isopentenyl-diphosphate delta isomerase 1	-0.30	0.046
11757814_s_at	SLC25A30	solute carrier family 25, member 30	-0.41	0.046
11718418_a_at	TST	thiosulfate sulfurtransferase (rhodanese)	0.43	0.047
11754083_a_at	BSG	basigin (Ok blood group)	-0.41	0.047
11740020_at	OTC	ornithine carbamoyltransferase	0.21	0.047
11727393_a_at	COX15	COX15 homolog, cytochrome c oxidase assembly protein (yeast)	0.21	0.048
11731831_x_at	DHDPSL	dihydrodipicolinate synthase-like, mitochondrial	0.28	0.048
11723707_a_at	AIFM2	apoptosis-inducing factor, mitochondrion-associated, 2	0.34	0.048
11754487_x_at	C5orf33	chromosome 5 open reading frame 33	0.36	0.048
11723467_a_at	NDUFA3	NADH dehydrogenase (ubiquinone) 1 alpha subcomplex, 3, 9kDa	0.18	0.049
11754240_a_at	MRPS5	mitochondrial ribosomal protein S5	0.23	0.049
11748456_a_at	MCF2L	MCF.2 cell line derived transforming sequence-like	-0.26	0.049
11718685_a_at	TXNRD2	thioredoxin reductase 2	-0.85	0.050

11731082_at	RNF144B	ring finger protein 144B	-0.50	0.050
11717296_s_at	LRPPRC	leucine-rich PPR-motif containing	-0.34	0.050
11726975_x_at	PERP	PERP, TP53 apoptosis effector	0.29	0.050

Supplementary Table S3: Significantly altered genes (nominal $p<0.05$) associated with glucose metabolism based on KEGG glycolysis/gluconeogenesis gene set.

ID	Gene Symbol	Gene Title	Log2 FC	P-Value
11754184_a_at	ALDH1A3	aldehyde dehydrogenase 1 family, member A3	-0.93	0.003
11751154_x_at	ALDOC	aldolase C, fructose-bisphosphate	-0.47	0.008
11752103_a_at	HK1	hexokinase 1	-0.49	0.010
11718291_a_at	HK1	hexokinase 1	-0.47	0.011
11748423_a_at	PGM1	phosphoglucomutase 1	-0.65	0.011
11756127_a_at	PGM1	phosphoglucomutase 1	-0.69	0.012
11731491_a_at	HK1	hexokinase 1	-0.46	0.013
11747079_x_at	ALDOC	aldolase C, fructose-bisphosphate	-0.43	0.014
11718292_s_at	HK1	hexokinase 1	-0.44	0.016
11751153_a_at	ALDOC	aldolase C, fructose-bisphosphate	-0.35	0.018
11724462_x_at	ALDOC	aldolase C, fructose-bisphosphate	-0.43	0.021
11753466_a_at	ACSS2	acyl-CoA synthetase short-chain family member 2	-0.95	0.021
11730931_at	ACSS3	acyl-CoA synthetase short-chain family member 3	0.34	0.024
11724461_a_at	ALDOC	aldolase C, fructose-bisphosphate	-0.47	0.025
11752342_x_at	PKM2	pyruvate kinase, muscle	-0.26	0.032
11744437_a_at	ADH6	alcohol dehydrogenase 6 (class V)	0.30	0.034
11752166_x_at	PKM2	pyruvate kinase, muscle	-0.35	0.036
11759049_at	ACSS3	acyl-CoA synthetase short-chain family member 3	0.31	0.038

Supplementary Table S4: Overlap between significantly decreased genes (nominal p<0.05) and the molecular signature database C3 (motif) gene set.

Gene Set Name [# Genes (K)]	Description	# Genes in Overlap (k)	p-value	FDR q-value
CAGGTG_V$E12_Q6 [2485]	motif CAGGTG which matches TCF3: transcription factor 3 (E2A immunoglobulin enhancer binding factors E12/E47)	228	4.68 e^{-65}	3.91 e^{-62}
GGGCGGR_V$SP1_Q6[2940]	SP1: Sp1 transcription factor	213	9.41 e^{-44}	3.93 e^{-41}
AACTTT_UNKNOWN [1890]	motif AACTTT. Motif does not match any known transcription factor	165	1.42 e^{-43}	3.96 e^{-41}
TGGAAA_V$NFAT_Q4_01 [1896]	motif TGGAAA which matches NFAT NFATC	163	3.48 e^{-42}	7.27 e^{-40}
TGANTCA_V$AP1_C [1121]	motif TGANTCA which matches JUN: jun oncogene	114	4.59 e^{-36}	7.67 e^{-34}
GGGAGGRR_V$MAZ_Q6 [2274]	motif GGGAGGRR which matches MAZ: MYC-associated zinc finger protein (purine-binding transcription factor)	167	1.11 e^{-34}	1.55 e^{-32}
CTTTGT_V$LEF1_Q2 [1972]	motif CTTTGT which matches LEF1: lymphoid enhancer-binding factor 1	150	9.36 e^{-33}	1.12 e^{-30}
TTGTTT_V$FOXO4_01 [2061]	motif TTGTTT which matches MLLT7: myeloid/lymphoid or mixed-lineage leukemia (trithorax homolog, Drosophila); translocated to, 7	149	3.68 e^{-30}	3.85 e^{-28}
CAGCTG_V$AP4_Q5 [1524]	motif CAGCTG which matches REPIN1: replication initiator 1	122	1.21 e^{-28}	1.13 e^{-26}
RYTTCCTG_V$ETS2_B [1085]	motif RYTTCCTG which matches ETS2: v-ets erythroblastosis virus E26 oncogene homolog 2 (avian)	97	1.97 e^{-26}	1.65 e^{-24}
TGTTTAC,MIR-30A-5P, MIR-30C,MIR-30D, MIR-30B,MIR-30E-5P [579]	Targets of MicroRNA TGTTTAC,MIR-30A-5P,MIR-30C,MIR-30D,MIR-30B,MIR-30E-5P	65	2.94 e^{-23}	2.24 e^{-21}
GGGTGGRR_V$PAX4_03 [1294]	motif GGGTGGRR which matches PAX4: paired box gene 4	101	5.41 e^{-23}	3.67 e^{-21}
CAGTATT,MIR-200B,MIR-200C,MIR-429 [469]	Targets of MicroRNA CAGTATT,MIR-200B,MIR-200C,MIR-429	58	5.7 e^{-23}	3.67 e^{-21}
TTANTCA_UNKNOWN [952]	motif TTANTCA. Motif does not match any known transcription factor	84	1.23 e^{-22}	7.33 e^{-21}
TTTGCAC,MIR-19A,MIR-19B [516]	Targets of MicroRNA TTTGCAC,MIR-19A,MIR-19B	60	2.44 e^{-22}	1.36 e^{-20}
GCANCTGNY_V$MYOD_Q6 [924]	motif GCANCTGNY which matches MYOD1: myogenic differentiation 1	82	2.79 e^{-22}	1.46 e^{-20}
GTGCCTT,MIR-506 [727]	Targets of MicroRNA GTGCCTT,MIR-506	71	9.18 e^{-22}	4.52 e^{-20}
CTTTGA_V$LEF1_Q2 [1232]	motif CTTTGA which matches LEF1: lymphoid enhancer-binding factor 1	95	2.6 e^{-21}	1.21 e^{-19}
TGCCTTA,MIR-124A [552]	Targets of MicroRNA TGCCTTA,MIR-124A	59	3.56 e^{-20}	1.57 e^{-18}
WGGAATGY_V$TEF1_Q6 [378]	motif WGGAATGY which matches TEAD1: TEA domain family member 1 (SV40 transcriptional enhancer factor)	47	5.84 e^{-19}	2.44 e^{-17}
TATAAA_V$TATA_01 [1296]	motif TATAAA which matches TAF TATA	93	8.23 e^{-19}	3.27 e^{-17}
CTGCAGY_UNKNOWN [765]	motif CTGCAGY. Motif does not match any known transcription factor	67	4.07 e^{-18}	1.55 e^{-16}
TGGNNNNNNKCCAR_UNKNOWN [424]	motif TGGNNNNNNKCCAR. Motif does not match any known transcription factor	48	1.16 e^{-17}	4.23 e^{-16}

GGGYGTGNY_UNKNOWN [664]	motif GGGYGTGNY. Motif does not match any known transcription factor	61	$1.38\ e^{-17}$	$4.81\ e^{-16}$
ACTGTGA,MIR-27A,MIR-27B [474]	Targets of MicroRNA ACTGTGA,MIR-27A,MIR-27B	50	$4.63\ e^{-17}$	$1.55\ e^{-15}$
RGAGGAARY_V$PU1_Q6 [502]	motif RGAGGAARY which matches SPI1: spleen focus forming virus (SFFV) proviral integration oncogene spi1	51	$1.08\ e^{-16}$	$3.46\ e^{-15}$
TGACCTY_V$ERR1_Q2 [1043]	motif TGACCTY which matches ESRRA: estrogen-related receptor alpha	77	$1.99\ e^{-16}$	$6.17\ e^{-15}$
TAGCTTT,MIR-9 [236]	Targets of MicroRNA TAGCTTT,MIR-9	34	$4.92\ e^{-16}$	$1.39\ e^{-14}$
TGGTGCT,MIR-29A,MIR-29B,MIR-29C [521]	Targets of MicroRNA TGGTGCT,MIR-29A,MIR-29B,MIR-29C	51	$4.96\ e^{-16}$	$1.39\ e^{-14}$
TGCACTT,MIR-519C,MIR-519B,MIR-519A [448]	Targets of MicroRNA TGCACTT,MIR-519C,MIR-519B,MIR-519A	47	$5\ e^{-16}$	$1.39\ e^{-14}$
YTATTTTNR_V$MEF2_02 [697]	motif YTATTTTNR which matches MEF2A: MADS box transcription enhancer factor 2, polypeptide A (myocyte enhancer factor 2A)	60	$5.18\ e^{-16}$	$1.4\ e^{-14}$
TGCCAAR_V$NF1_Q6 [722]	motif TGCCAAR which matches NF1: neurofibromin 1 (neurofibromatosis, von Recklinghausen disease, Watson disease)	61	$6.98\ e^{-16}$	$1.82\ e^{-14}$
TGACAGNY_V$MEIS1_01 [827]	motif TGACAGNY which matches MEIS1: Meis1, myeloid ecotropic viral integration site 1 homolog (mouse)	66	$7.29\ e^{-16}$	$1.85\ e^{-14}$
GCACTTT,MIR-17-5P, MIR-20A,MIR-106A, MIR-106B,MIR-20B,MIR-519D [595]	Targets of MicroRNA GCACTTT,MIR-17-5P,MIR-20A,MIR-106A,MIR-106B,MIR-20B,MIR-519D	54	$1.7\ e^{-15}$	$4.18\ e^{-14}$
CTTTAAR_UNKNOWN [972]	motif CTTTAAR. Motif does not match any known transcription factor	71	$5.22\ e^{-15}$	$1.25\ e^{-13}$
TGAATGT,MIR-181A, MIR-181B,MIR-181C, MIR-181D [484]	Targets of MicroRNA TGAATGT,MIR-181A,MIR-181B,MIR-181C,MIR-181D	47	$9.47\ e^{-15}$	$2.2\ e^{-13}$
TAATTA_V$CHX10_01 [810]	motif TAATTA which matches VSX1: visual system homeobox 1 homolog, CHX10-like (zebrafish)	63	$1.1\ e^{-14}$	$2.48\ e^{-13}$
TTGCCAA,MIR-182 [327]	Targets of MicroRNA TTGCCAA,MIR-182	38	$1.23\ e^{-14}$	$2.69\ e^{-13}$
TGACATY_UNKNOWN [665]	motif TGACATY. Motif does not match any known transcription factor	56	$1.26\ e^{-14}$	$2.69\ e^{-13}$
GGATTA_V$PITX2_Q2 [587]	motif GGATTA which matches PITX2: paired-like homeodomain transcription factor 2	52	$1.51\ e^{-14}$	$3.15\ e^{-13}$
V$ER_Q6_02 [252]	motif NAGGTCANNNY which matches ESR1: estrogen receptor 1	32	$1.3\ e^{-13}$	$2.65\ e^{-12}$
V$SRF_C [211]	motif DCCWTATATGGNCWN which matches SRF: serum response factor (c-fos serum response element-binding transcription factor)	29	$2.37\ e^{-13}$	$4.72\ e^{-12}$
V$AMEF2_Q6 [259]	motif CKGDYTAAAAATAACYMM. Motif does not match any known transcription factor	32	$2.79\ e^{-13}$	$5.42\ e^{-12}$
AAGCACA,MIR-218 [398]	Targets of MicroRNA AAGCACA,MIR-218	40	$3.06\ e^{-13}$	$5.82\ e^{-12}$
V$AP1_Q6_01 [264]	motif NTGACTCAN which matches JUN: jun oncogene	32	$4.73\ e^{-13}$	$8.8\ e^{-12}$
CAGCTTT,MIR-320 [256]	Targets of MicroRNA CAGCTTT,MIR-320	31	$1.12\ e^{-12}$	$2.04\ e^{-11}$
RNGTGGGC_UNKNOWN [766]	motif RNGTGGGC. Motif does not match any known transcription factor	57	$1.26\ e^{-12}$	$2.24\ e^{-11}$
WTGAAAT_UNKNOWN [616]	motif WTGAAAT. Motif does not match any known transcription factor	50	$1.31\ e^{-12}$	$2.29\ e^{-11}$
V$AP1_C [275]	motif NTGASTCAG which matches JUN: jun oncogene	32	$1.45\ e^{-12}$	$2.47\ e^{-11}$
ACCAAAG,MIR-9 [499]	Targets of MicroRNA ACCAAAG,MIR-9	44	$1.87\ e^{-12}$	$3.08\ e^{-11}$

Supplementary Table S5: Significantly altered genes (nominal $p<0.05$) in or associated with the p53 family.

Id	Gene Symbol	Gene Title	Log2 FC	P-Value
11749789_a_at	TP53I3	tumor protein p53 inducible protein 3	0.47	0.02
11735724_a_at	TP53BP1	tumor protein p53 binding protein 1	0.70	0.02
11752567_a_at	TP63	tumor protein p63	-0.24	0.02
11736193_a_at	RRM2B	ribonucleotide reductase M2 B (TP53 inducible)	-0.49	0.02
11736192_at	RRM2B	ribonucleotide reductase M2 B (TP53 inducible)	-0.41	0.02
11718808_a_at	TP53BP1	tumor protein p53 binding protein 1	0.62	0.03
11735725_x_at	TP53BP1	tumor protein p53 binding protein 1	0.64	0.03
11736821_x_at	TP73	tumor protein p73	-0.24	0.03
11754177_a_at	RRM2B	ribonucleotide reductase M2 B (TP53 inducible)	-0.36	0.04
11726975_x_at	PERP	PERP, TP53 apoptosis effector	0.29	0.05
11751537_x_at	TP53	tumor protein p53	0.23	0.05

Supplementary Table S6: Official gene symbols and names with primer sequences used for qRT-PCR.

Gene Symbol	Gene Name	Forward Primer 5'-3'	Reverse Primer 5'-3'
ACTB	actin, beta	CATGTACGTTGCTATCCAGGC	CTCCTTAATGTCACGCACGAT
ALDOA	aldolase A, fructose-bisphosphate	GGCCATGCTTGCACTCAGAAGTTT	AATGGCATTGAGGTTGATGGACGC
ATP5A1	ATP synthase, H+ transporting, mitochondrial F1 complex, alpha subunit 1, cardiac muscle	TGCAAGGAACTTCCATGCCTC	CGCCCAGTTTCTTCAAGATCAA
CAT	catalase	TGGGATCTCGTTGGAAATAACAC	TCAGGACGTAGGCTCCAGAAG
CYC1	cytochrome c-1	CTTCGCGGGGTAGTGTTGG	GGCCAGACTTCGACGACAA
CYCS	cytochrome c, somatic	CTTTGGGCGGAAGACAGGTC	TTATTGGCGGCTGTGTAAGAG
DNM1L	dynamin 1-like	TCACCCGGAGACCTCTCATT	TCTTCTGCTTCCACCCCATT
E2F1	E2F transcription factor 1	GGCCGCCATCCAGGAAAAGGTG	GACGACACCGTCAGCCGAGT
E2F4	E2F transcription factor 4, p107/p130-binding	CACCACCAAGTTCGTGTCCC	GCGTACAGCTAGGGTGTCA
ESRRA	estrogen-related receptor alpha	AAGACAGCAGCCCCAGTGAA	ACACCCAGCACCAGCACCT
ESRRG	estrogen-related receptor gamma	GCCCTCACTACACTGTGTGAC	CCTGCTAATTTGGACTGGTCTT
FIS1	fission, mitochondrial 1	GTGGAGGACCTGCTGAAGTTT	GCACGATGCCTTTACGGATG
GABPA	GA binding protein transcription factor alpha subunit	AGGAAGTGGAGGGACTGATCC	GGCGCGTAGGTTTGTTCTAC
GABPB1	GA binding protein transcription factor, beta subunit 1	TGCCAGATGGACAACAAGTATT	GCAAAGCACACCGGGTAAAA
GABPB2	GA binding protein transcription factor, beta subunit 2	AATCTTACTCGCGGCGCCTT	CAAAGACATCTCCTCGTCCGGG
GAPDH	glyceraldehyde-3-phosphate dehydrogenase	CCAGGGCTGCTTTTAACTC	GCTCCCCCCTGCAAATGA
GPX1	glutathione peroxidase 1	TATCGAGAATGTGGCGTCCC	TCTTGGCGTTCTCCTGATGC
HK1	hexokinase 1	CCAACATTCGTAAGGTCCATTCC	CCTCGGACTCCATGTGAACATT
HK2	hexokinase 2	GAGCCACCACTCACCCTACT	CCAGGCATTCGGCAATGTG
INF2	inverted formin, FH2 and WH2 domain containing	CTCACCGGCTCGGCAAGAT	GCCGGAGTAGTTGACCACAG
MFF	mitochondrial fission factor	TGACCACGAACACTCTTCCG	GTGCTGGATTGAGAGCCACT
MFN1	mitofusin 1	GCCTCCTCTCCGCCTTTAAC	TATGCTAAGTCTCCGCTCCAA
MFN2	mitofusin 2	GAGGCGTAAGGAGTAGGCGG	AGGGACATTGCGCTTCACCT
MIEF1	mitochondrial elongation factor 1	AACGGCAAATTGGGGAGGAT	GCCATTGCTCATCTGAGGCT
mt-ND1	mitochondrially encoded NADH dehydrogenase 1	ATGGCCAACCTCCTACTCCTCATT	TTATGGCGTCAGCGAAGGGTTGTA
mt-ND2	mitochondrially encoded NADH dehydrogenase 2	CCATCTTTGCAGGCACACTCATCA	ATTATGGATGCGGTTGCTTGCGTG
NDUFV1	NADH:ubiquinone oxidoreductase core subunit V1	AGGATGAAGACCGGATTTTCAC	CAGTCACCTCGACTCAGGGA
OPA1	optic atrophy 1 (autosomal dominant)	TGGTCTAACAAGCTCTCAGTGTGG	ACCGGTTGACCATGGAGTACAACT
PKM2	pyruvate kinase, muscle isoform a	AAGGACCTGAGATCCGAACTG	GCGTTATCCAGCGTGATTTTGA
PPARGC1A	peroxisome proliferator-activated receptor gamma, coactivator 1 alpha	TCTGAGTCTGTATGGAGTGACAT	CCAAGTCGTTCACATCTAGTTCA
PPARGC1B	peroxisome proliferator-activated receptor gamma, coactivator 1 beta	GATGCCAGCGACTTTGACTC	ACCCACGTCATCTTCAGGGA
PPRC1	peroxisome proliferator-activated receptor gamma, coactivator-related 1	CAAGCGCCGTATGGGACTTT	GGAGGCATCCATGTAGCTCT
RB1	retinoblastoma 1	CCTCATGCTGTTCAGGAGACAT	GGCTTCGAGGAATGTGAGGT
SDHA	succinate dehydrogenase complex subunit A, flavoprotein (Fp)	CAAACAGGAACCCGAGGTTTT	CAGCTTGGTAACACATGCTGTAT
SIRT1	sirtuin 1	AGCTAGGACCATTACTGCCAGAGA	AGCACACAAACATCATGCAAATGG
SIRT2	sirtuin 2	TCATGGGTACCTCCTTGCAG	CTCCAGCTCCTTCTTCCATCC
SIRT3	sirtuin 3	ACCCAGTGGCATTCCAGAC	GGCTTGGGGTTGTGAAAGAAG
SLC2A1	solute carrier family 2 (facilitated glucose transporter), member 1	GGCCAAGAGTGTGCTAAAGAA	ACAGCGTTGATGCCAGACAG
SLC2A4	solute carrier family 2 (facilitated glucose transporter), member 4	GTCGGGCTTCCAACAGATAG	AGCACCGCAGAGAACACAG
SOD1	superoxide dismutase 1, soluble	GGTGGGCCAAAGGATGAAGAG	CCACAAGCCAAACGACTTCC
SOD2	superoxide dismutase 2, mitochondrial	GGAAGCCATCAAACGTGACTT	CCCGTTCCTTATTGAAACCAAGC
SP1	transcription factor SP1	GGTTCGCTTGCCTCGTCAG	GGAGTGATCTTGGTCGCTCAT
TFAM	transcription factor A, mitochondrial	AACACTGCTTGGAAAACCAAA	CAGCTTTTCCTGCGGTGAAT
UCP1	uncoupling protein 1 (mitochondrial, proton carrier)	AGCGGCAAATCAGCTCCGCC	ACGACCTCTGTGGGTTGCCC
YY1	YY1 transcription factor	GCTTGCCCTCATAAAGGCTG	ACATTCTGCACAGACGTGGA
